# Supplementary material for: Designing lithium halide solid electrolytes
Source: Nat Commun. 2024 Feb 5;15:1050. doi: 10.1038/s41467-024-45258-3 (PMC10844219; doi:10.1038/s41467-024-45258-3)
Supplement: Supplementary file 1 — Supplementary information [file 41467_2024_45258_MOESM1_ESM.pdf]

# Supplementary information

# Designing lithium halide solid electrolytes

Qidi Wang[1], Yunan Zhou[2], Xuelong Wang[3], Hao Guo[4], Shuiping Gong[5], Zhenpeng Yao[5], Fangting Wu[2], Jianlin Wang[6], Swapna Ganapathy[1], Xuedong Bai[6], Baohua Li[2], Chenglong Zhao[1], Jürgen Janek[7] , Marnix Wagemaker[1]

[1]Department of Radiation Science and Technology, Delft University of Technology, Delft 2629JB, the Netherlands.

[2]Shenzhen Key Laboratory on Power Battery Safety and Shenzhen Geim Graphene Center, School of Shenzhen International Graduate, Tsinghua University, Guangdong 518055, China.

[3]Chemistry Division, Brookhaven National Laboratory, New York 11973, United States.

[4]Neutron Scattering Laboratory, Department of Nuclear Physics, China Institute of Atomic Energy, Beijing 102413, China.

[5]The State Key Laboratory of Metal Matrix Composites, School of Materials Science and Engineering, Center of Hydrogen Science, Innovation Center for Future Materials, Zhangjiang Institute for Advanced Study, Shanghai Jiao Tong University, Shanghai 200240, China.

[6]State Key Laboratory for Surface Physics, Institute of Physics, Chinese Academy of Sciences, Beijing 100190, China.

[7]Institute of Physical Chemistry, Center for Materials Research, Justus-Liebig-University Giessen, Giessen D-35392, Germany.

## Table of Contents

**Supplementary Notes 1-3..........................................................................Pages 3-5**

**Supplementary Figures 1-40.....................................................................Pages 6-45**

**Supplementary Tables 1-15.......................................................................Pages 46-60**

**Supplementary References.......................................................................Pages 61-62**

## Supplementary Notes

### Supplementary Note 1

The Li-ion metal halides are a larger family of compounds, having high compositional diversity. As for the use of SEs, such elements should not undergo redox reactions during the charge/discharge process. This leads to about 32 different Me ions that can participate in the formation of halide compounds within about 11 different space groups by screening the Li-containing halides from the inorganic crystal structure database (Supplementary Figs. 1-15 and Table 1). These structures have the different formulas of $Li_2MeX_4$, $Li_3MeX_6$, $Li_2MeX_6$ and $LiMeX_6$ associated with Me ions with formal charge numbers 2, 3, 4 and 5, respectively. Generally, the $Li_2MeX_4$ compounds can be indexed into *Fd*-3*m*, *Cmmm* and *Pnma* structures with [$MeX_4$] and [$MeX_6$] polyhedra (Supplementary Figs. 9, 10 and 15). The $Li_2MeX_6$ compounds show *P*-3*m*1, *P*-$3_1m$, *C*2/*c*, *P*$2_1$/*c* and *C*2/*m* structures with [$MeX_6$] and [$MeX_8$] polyhedra (Supplementary Figs. 3-5 and 12). The $LiMeX_6$ compounds with pentavalent elements are found to show the in *R*-3 structure with [$MeX_6$] octahedra (Supplementary Fig. 11). By comparison, $Li_3MeX_6$ composition with trivalent elements can form most of these structures, including *P*-3*c*1, *P*-3*m*1, *Pnma*, *Pna*$2_1$, *C*2/*c* and *C*2/*m*, but only with [$MeX_6$] octahedron. This indicates the possibility of which $Li_{3+m}Me_{1+n}X_6$ compound can be formed in general by substituting/doping.

**Supplementary Note 2**

The bulk microscopic Li-ion diffusion mechanism is studied using solid-state $^7$Li NMR, which can be realized by measuring the temperature-dependent $^7$Li static spin-lattice relaxation (SLR) rate of $1/T_1$ in the laboratory frame using a saturation recovery experiment[1-3]. $T_1$ is the spin-lattice relaxation time that can be determined using a saturation recovery experiment. The change of $1/T_1$ is directly related to the spectral density function of Li-ion jump process[4,5], where measurements as a function of temperature can provide the jump frequency and activation energy. When the SLR rate reaches the maximum value as the function of temperature, $\omega_0\tau$ approximately equals unity, representing the optimum in energy transfer between the Li-ions and their environment[6,7]. Here $\tau$ is the correlation time between jumps and $\omega_0$ the natural Larmor frequency, 155.506*(2π) MHz for this measurement.

**Supplementary Note 3**

To evaluate the surface energies of the main facet orientations of $Li_3InCl_6$ and $Li_{2.8}In_{0.2}Sc_{0.2}Yb_{0.2}Lu_{0.2}Zr_{0.2}Cl_6$ materials, first-principles calculations were carried out to generate the surface structures. Models with different surface terminations are considered for each orientation. The surface energy of the main facets, (001), (020), (110), (-111), (021), (-202), (131), and (13-3), are calculated. Surface models of $Li_3InCl_6$ were generated by the Pymatgen program[4] with a thickness of the vacuum slab of 15 Å to prevent interaction between two neighboring slabs under the periodic conditions. To determine the most stable surface termination of the $Li_3InCl_6$ structure, we selected the (001) facet and calculated the surface energies for all different surface terminations. As shown in Supplementary Fig. 31, eight surface models with different terminated (001) facets were considered, where the $In^{3+}$ including termination is found to have the lowest surface energy. Assuming this is true for all facets, and to be consistent, the $In^{3+}$ terminations is applied for all facet orientations considered. To simulate the HE-SE structures, three surface models are generated for each orientation by randomly replacing $In^{3+}$ with $Sc^{3+}$, $Zr^{4+}$, $Lu^{3+}$ and $Yb^{3+}$ in an equiatomic manner, respectively. The process was repeated three times with the one with the lowest surface energy adopted. It should be noticed that all facets are $In^{3+}$ terminated to enable consistent comparison. Surface structures of the eight orientations for both $Li_3InCl_6$ and $Li_{2.8}In_{0.2}Sc_{0.2}Yb_{0.2}Lu_{0.2}Zr_{0.2}Cl_6$ are shown in Supplementary Fig. 32.

## Supplementary Figures

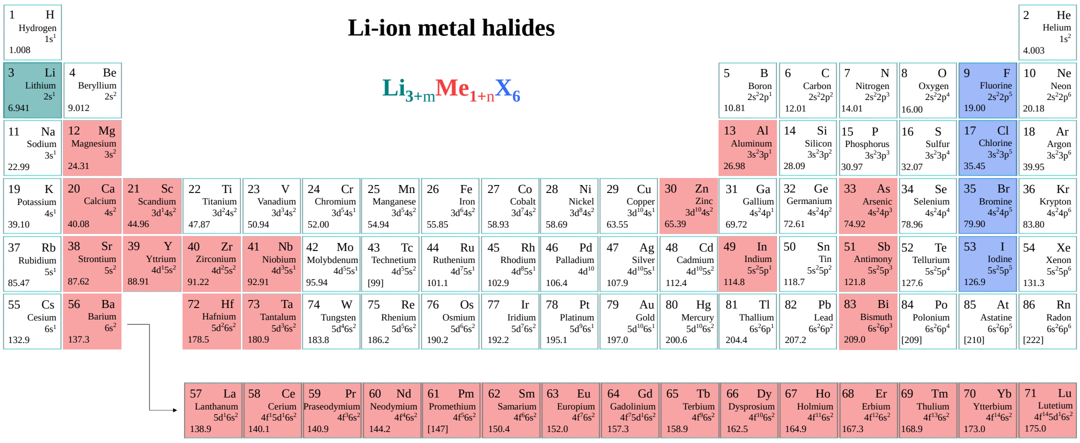


**Supplementary Fig. 1. Commonly used elements for formation of the Li-ion metal halide SEs.** The different color shows the possible distribution in $Li_{3+m}Me_{1+n}X_6$ composition.

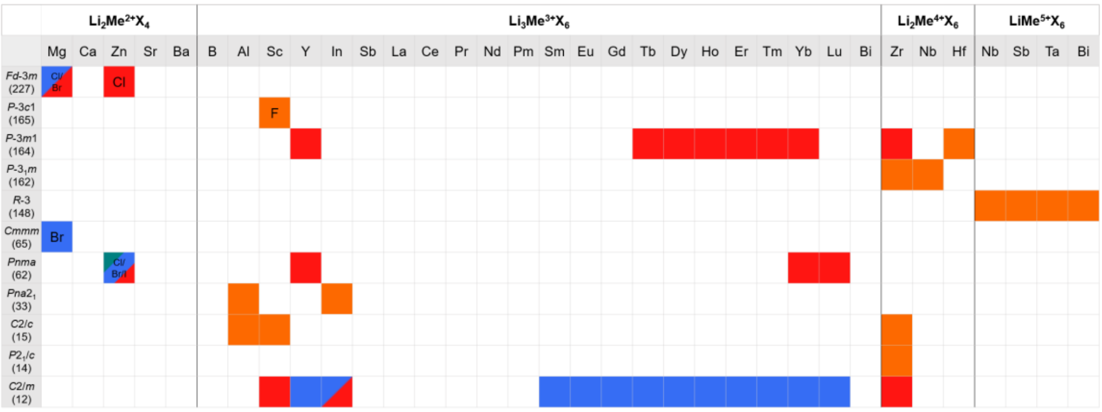


**Supplementary Fig. 2. Structural symmetry of Li-Me-X (X = F, Cl, Br and I) compositions.** Me is the metal ions with charge number of 2, 3, 4 and 5, which corresponds to the formal of $Li_2MeX_4$, $Li_3MeX_6$, $Li_2MeX_6$, and $LiMeX_6$, respectively. See Supplementary Table 1 for details.

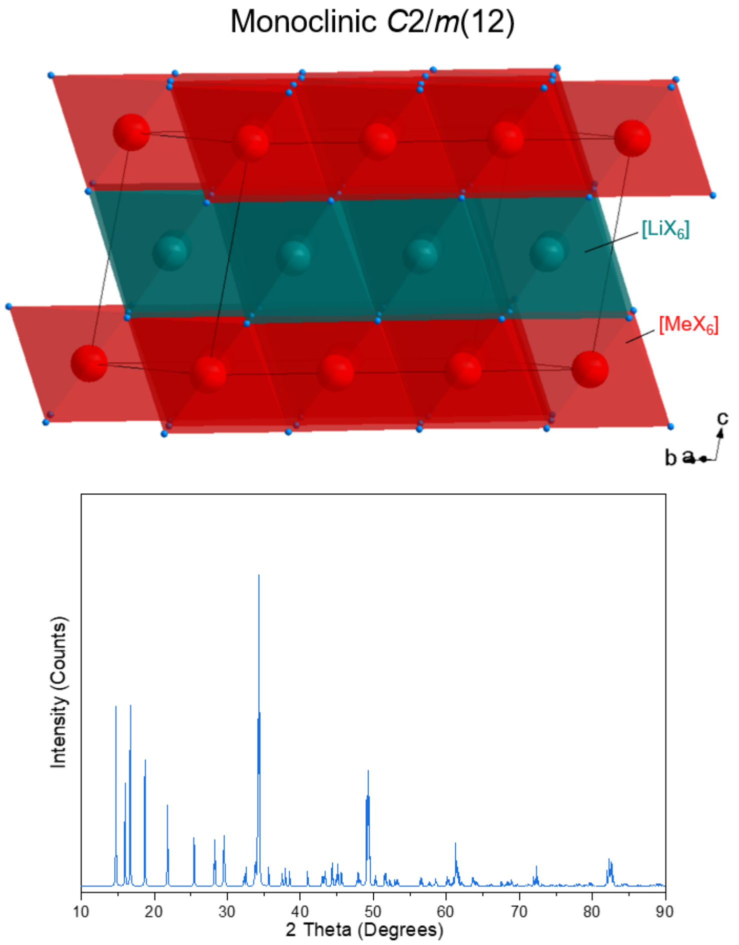


**Supplementary Fig. 3. Crystal structure and X-ray diffraction (XRD) pattern of monoclinic *C*2/*m*(12) Li-Me-X compound.** $Li_3InCl_6$ composition is selected to show the structure and diffraction information.

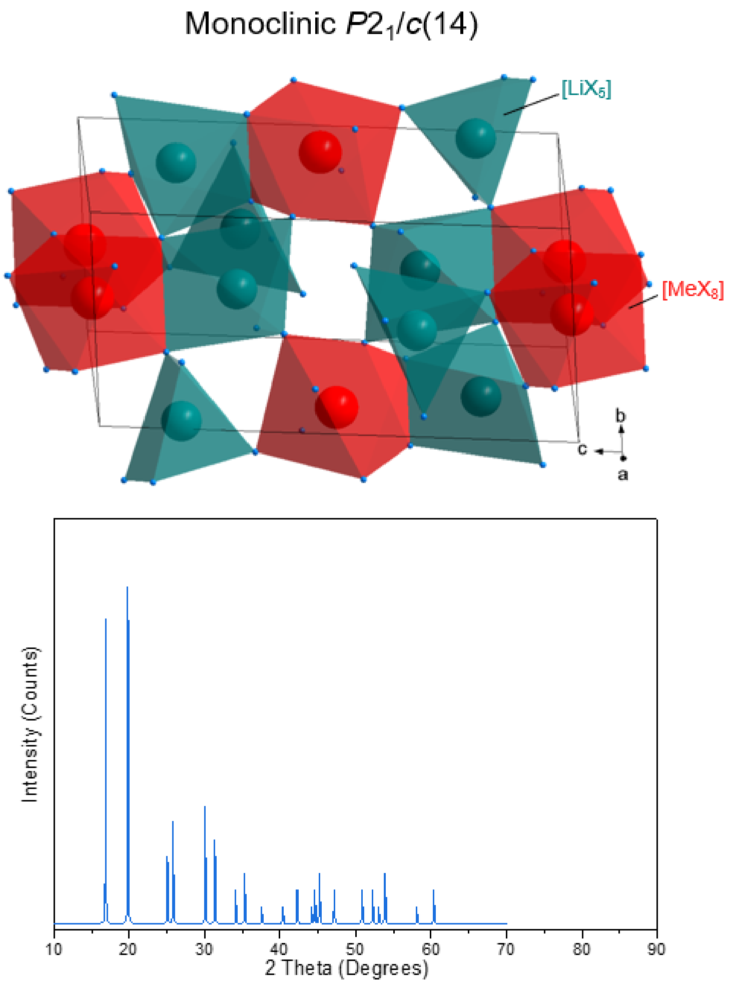


**Supplementary Fig. 4. Crystal structure and XRD pattern of monoclinic $P2_1/c$(14) Li-Me-X compound.** $Li_2ZrF_6$ composition is selected to show the structure and diffraction information.

Monoclinic *C*2/*c*(15)

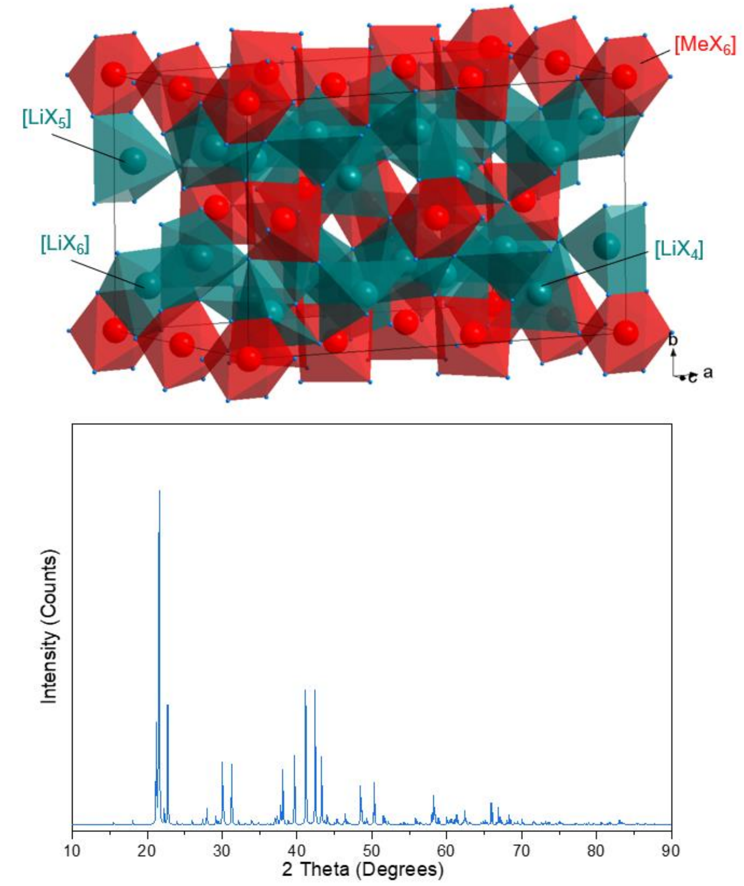


**Supplementary Fig. 5. Crystal structure and XRD pattern of monoclinic *C*2/*c*(15) Li-Me-X compound.** $Li_3AlF_6$ composition is selected to show the structure and diffraction information.

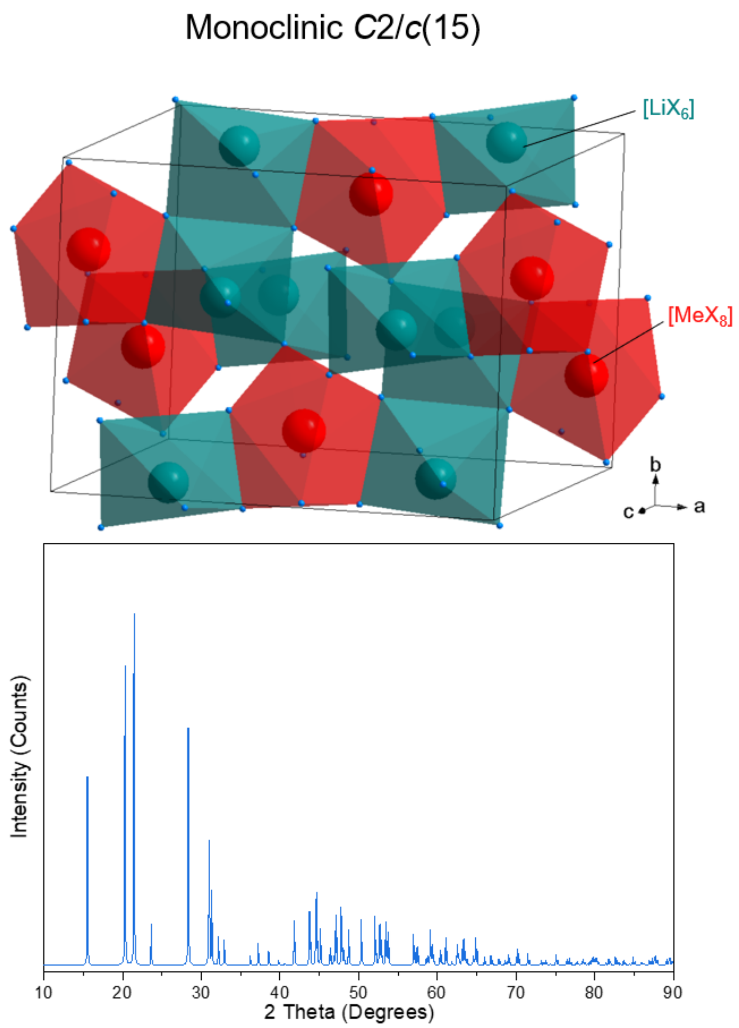


**Supplementary Fig. 6. Crystal structure and XRD pattern of monoclinic *C*2/*c*(15) Li-Me-X compound.** $Li_2ZrF_6$ composition is selected to show the structure and diffraction information.

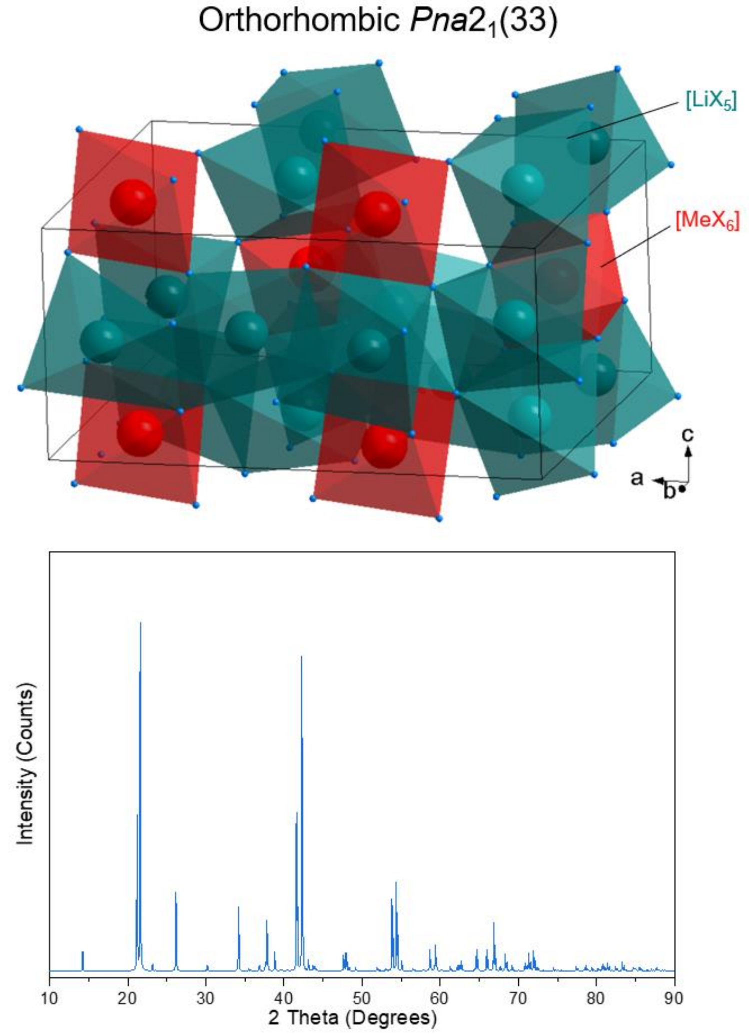


**Supplementary Fig. 7. Crystal structure and XRD pattern of orthorhombic *Pna*2$_1$(33) Li-Me-X compound.** $Li_3AlF_6$ composition is selected to show the structure and diffraction information.

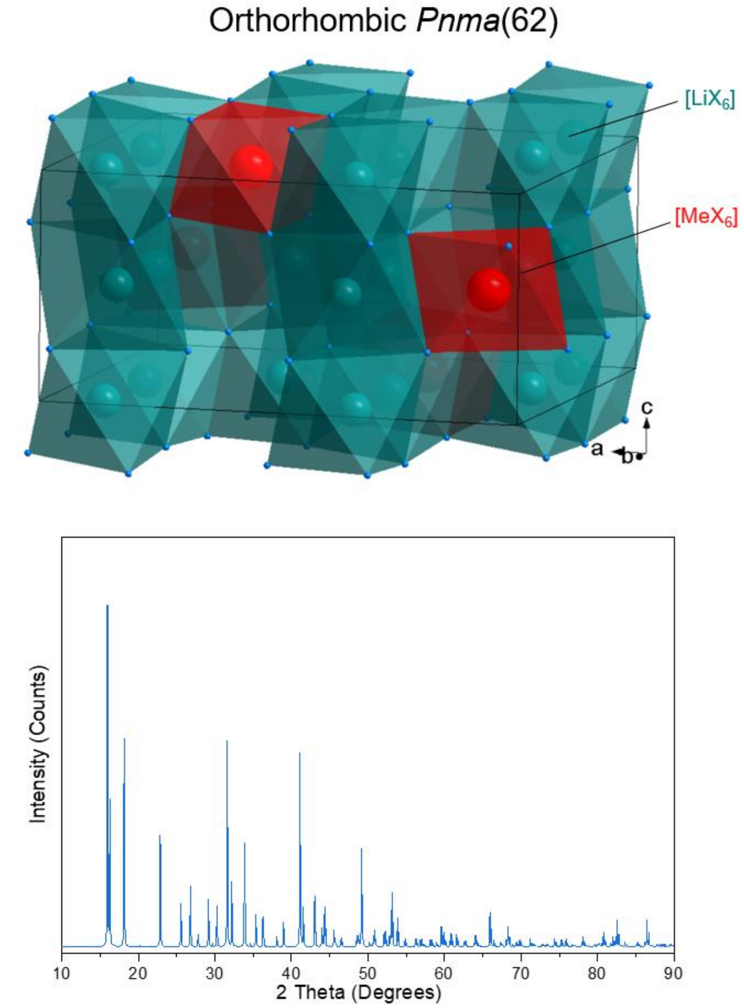


**Supplementary Fig. 8. Crystal structure and XRD pattern of orthorhombic *Pnma*(62) Li-Me-X compound.** $Li_3LuCl_6$ composition is selected to show the structure and diffraction information.

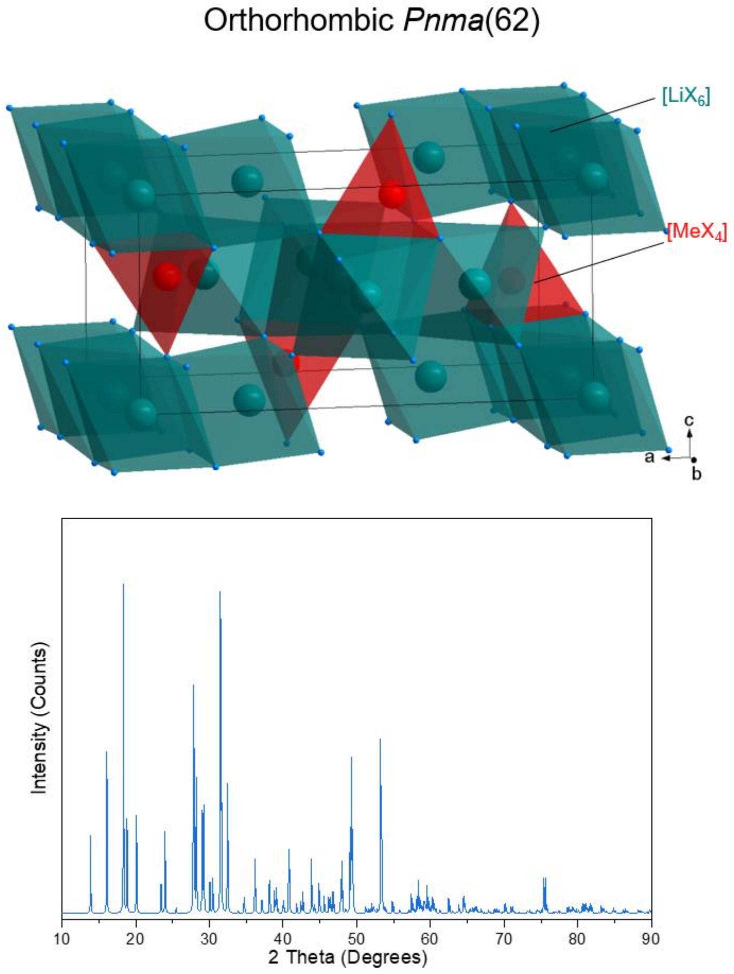


**Supplementary Fig. 9. Crystal structure and XRD pattern of orthorhombic *Pnma*(62) Li-Me-X compound.** $Li_2ZnCl_4$ composition is selected to show the structure and diffraction information.

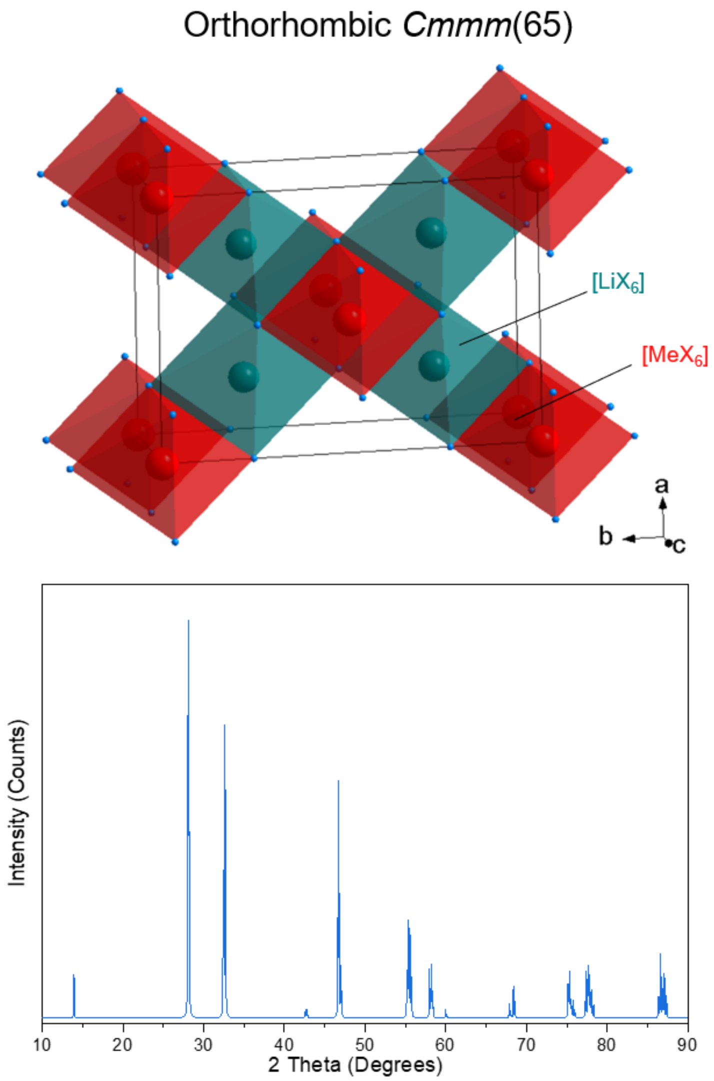


**Supplementary Fig. 10. Crystal structure and XRD pattern of orthorhombic *Cmmm*(65) Li-Me-X compound.** $Li_2MgBr_4$ composition is selected to show the structure and diffraction information.

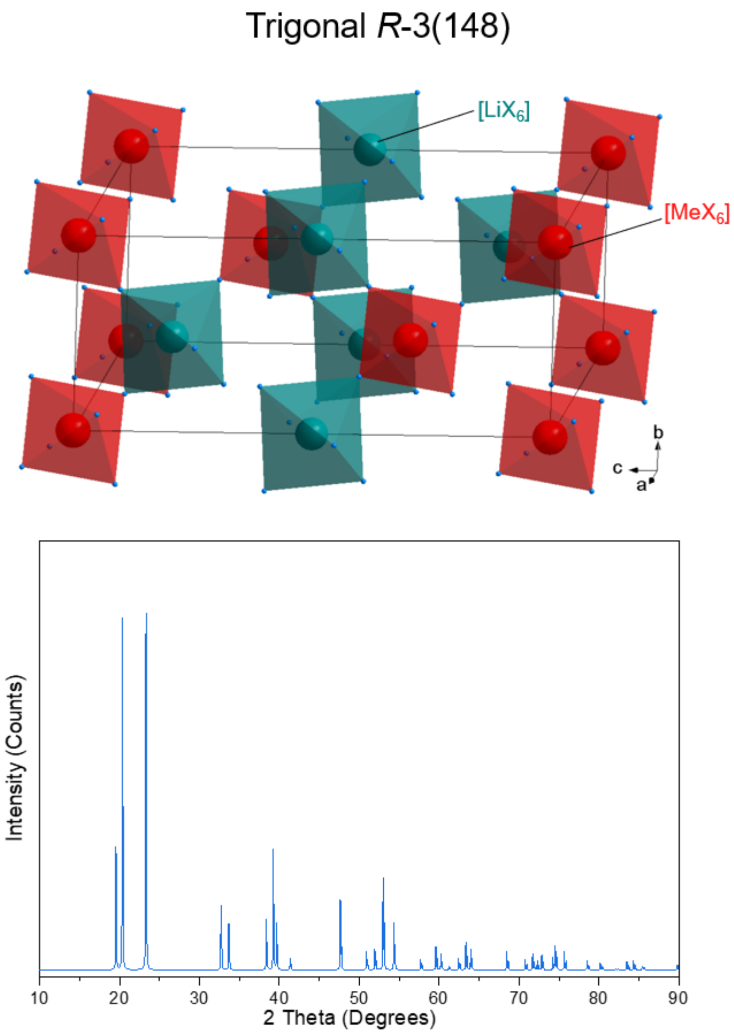


**Supplementary Fig. 11. Crystal structure and XRD pattern of trigonal *R*-3(148) Li-Me-X compound.** $LiTaF_6$ composition is selected to show the structure and diffraction information.

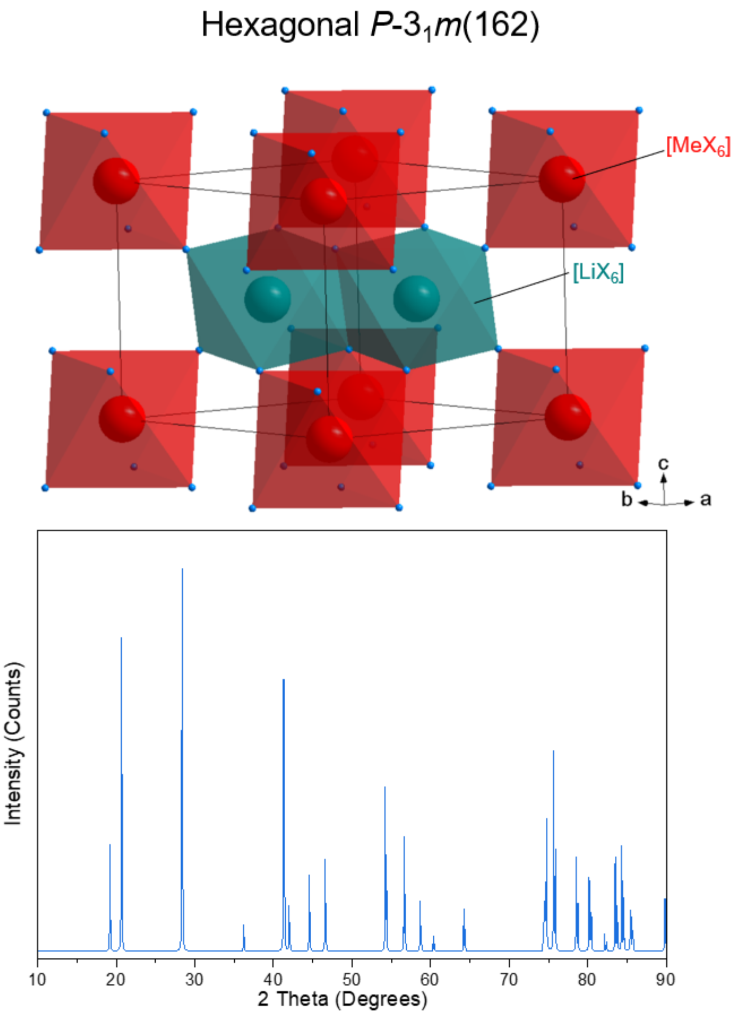


**Supplementary Fig. 12. Crystal structure and XRD pattern of hexagonal *P*-3$_1$*m*(162) Li-Me-X compound.** $Li_2NbF_6$ composition is selected to show the structure and diffraction information.

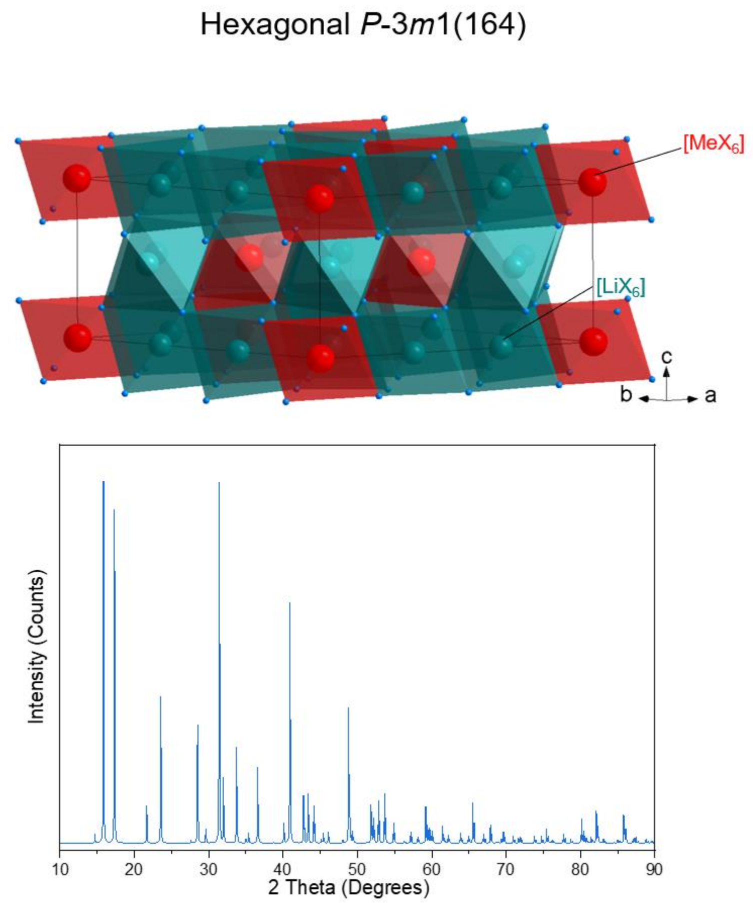


**Supplementary Fig. 13. Crystal structure and XRD pattern of hexagonal *P*-3*m*1(164) Li-Me-X compound.** $Li_3HoCl_6$ composition is selected to show the structure and diffraction information.

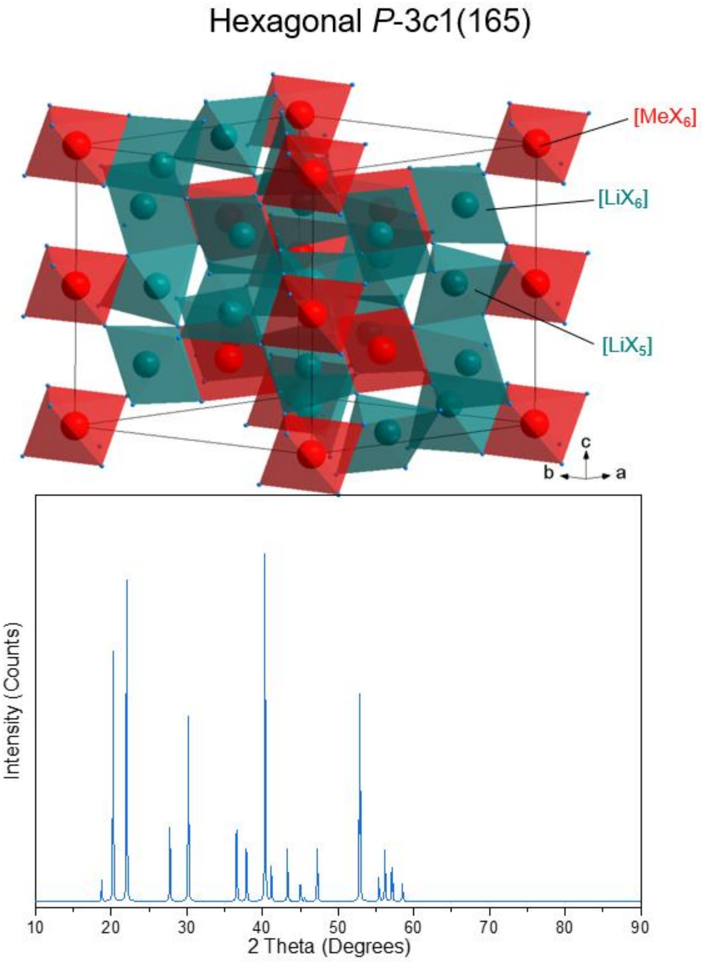


**Supplementary Fig. 14. Crystal structure and XRD pattern of hexagonal *P*-3*c*1(165) Li-Me-X compound.** $Li_3ScF_6$ composition is selected to show the structure and diffraction information.

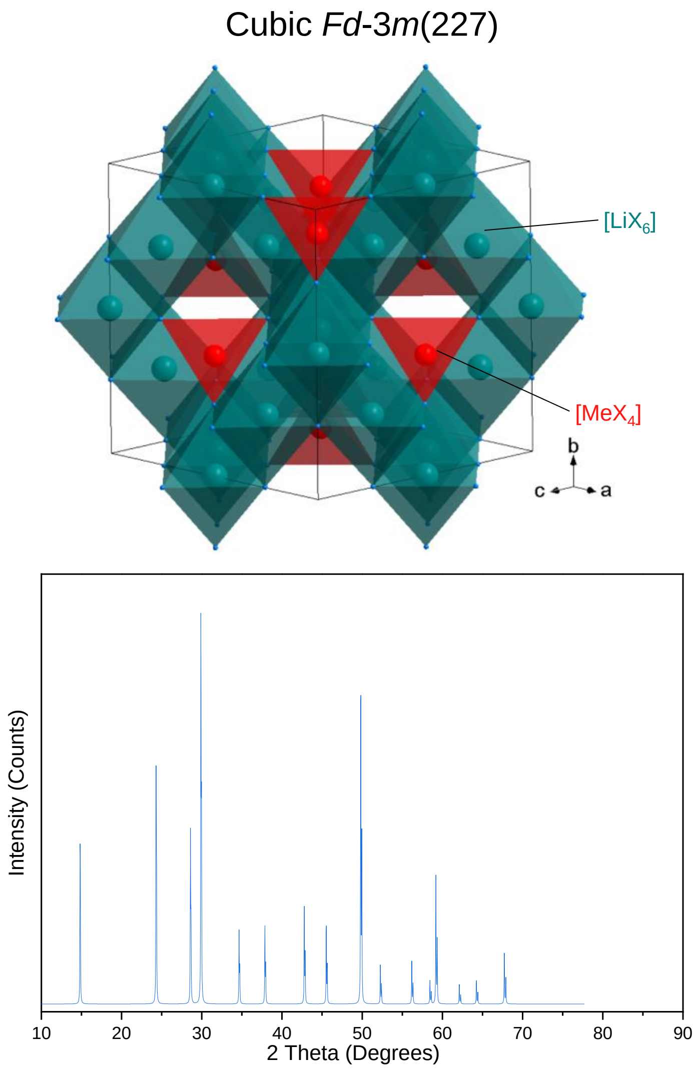


**Supplementary Fig. 15. Crystal structure and XRD pattern of cubic *Fd*-3*m*(227) Li-Me-X compound.** $Li_2ZnCl_4$ composition is selected to show the structure and diffraction information.

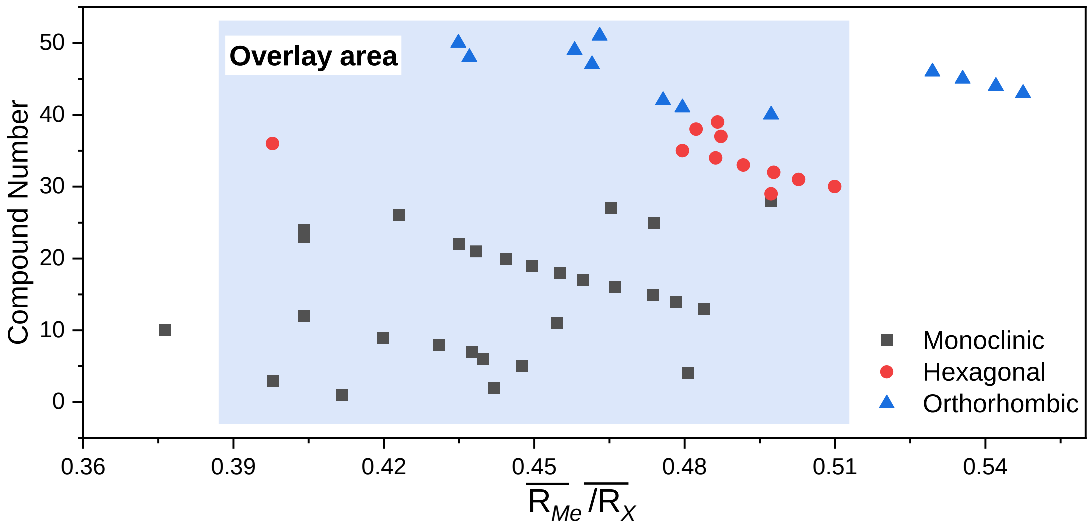


**Supplementary Fig. 16. Weighted average radius ratio between Me and X ions of representative halides with [$MeX_6$] packing.** As an attempt to use the radius ratio for compositions with more than one metal element, the weighted average radius was calculated and the ratio was plotted vs. compound number to show the phase distribution more clearly. However, the resulting plot shows a large overlapping area among the three phases. The compound number is corresponding to the order in the Supplementary Tables 2-4.

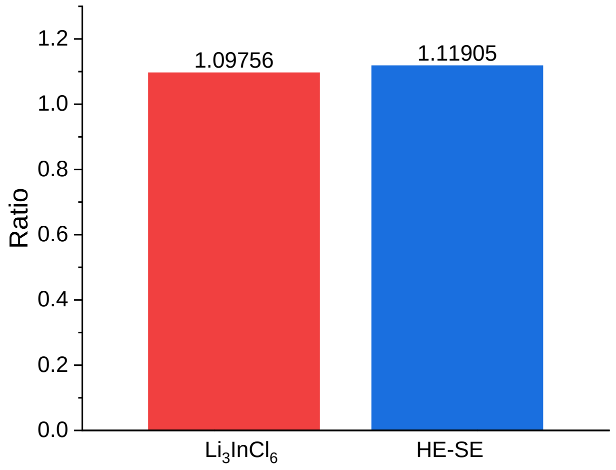


**Supplementary Fig. 17. The ratio of the interlayer distances of $d_{(X–Li–X)}$ and $d_{(X–Me–X)}$ for the layered halides.** The crystal information of $Li_3InCl_6$ is obtained from Ref. 8.

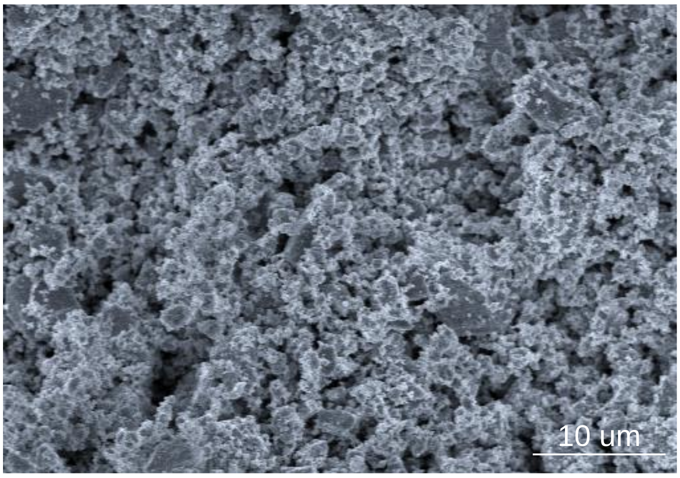


**Supplementary Fig. 18. Scanning electron microscopy (SEM) morphology of the as-prepared HE-SE.**

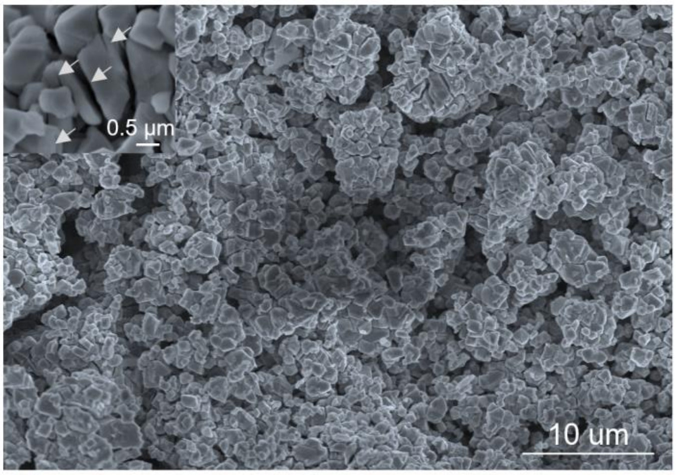


**Supplementary Fig. 19. SEM morphology of the general $Li_3InCl_6$.** $Li_3InCl_6$ is prepared using the same method as the HE-SE.

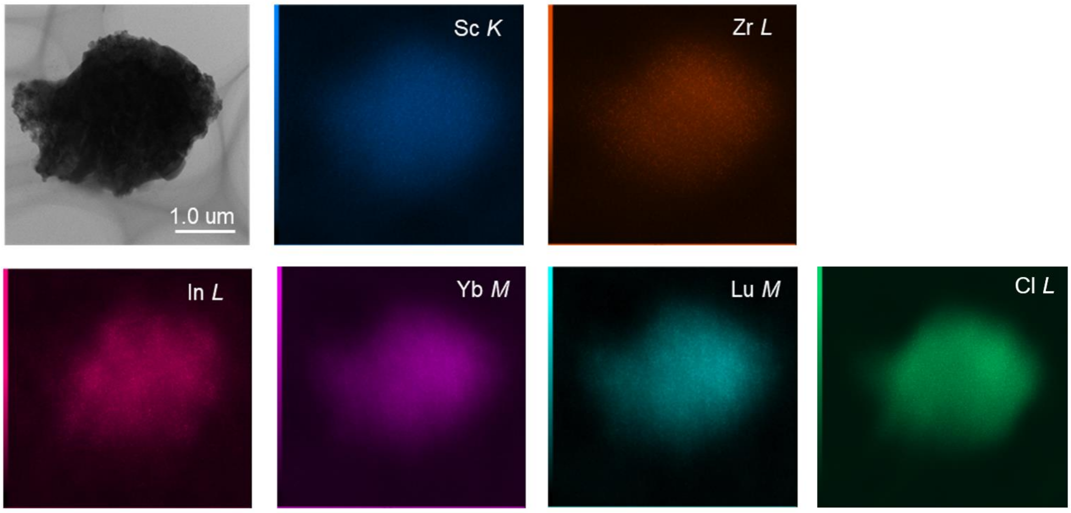


**Supplementary Fig. 20. Transmission electron microscopy (TEM) image and energy dispersive X-ray spectroscopy (EDS) mappings for as-prepared HE-SE.**

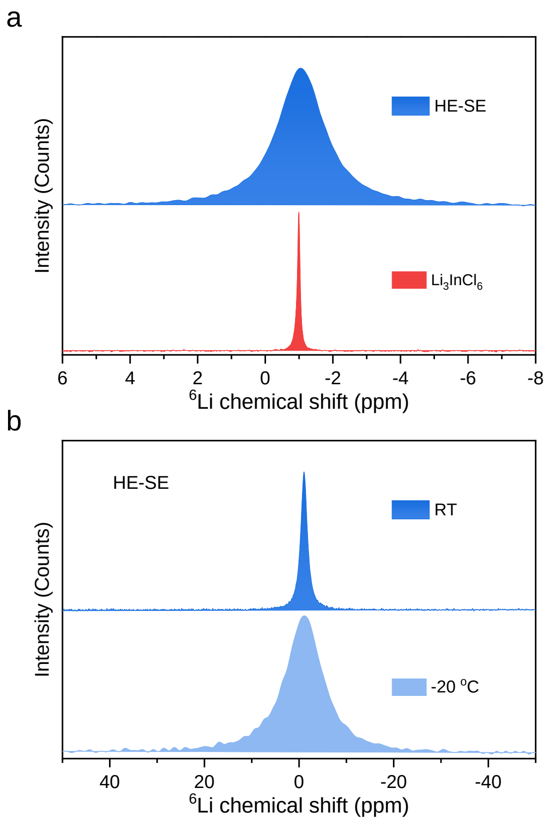


**Supplementary Fig. 21. Solid-state $^6$Li magic angle spinning (MAS) nuclear magnetic resonance (NMR) spectra of electrolytes. a**, Solid-state $^6$Li MAS-NMR spectra of the targeted HE-SE $Li_{2.8}In_{0.2}Sc_{0.2}Yb_{0.2}Lu_{0.2}Zr_{0.2}Cl_6$ material and the $Li_3InCl_6$ material at room temperature. **b**, Solid-state $^6$Li MAS-NMR spectra of the HE-SE material at different temperature. A broadened NMR spectrum is observed at low temperature, further suggesting a disordered distribution in Li-environments, which could be due to the complex composition in conjunction with the change in Li-ion site occupancies observed with diffraction.

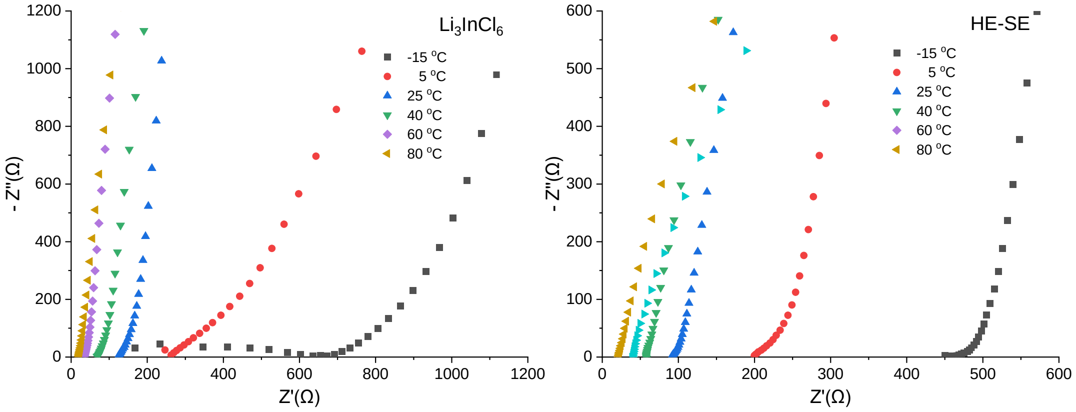


**Supplementary Fig. 22. Electrochemical impedance spectroscopy (EIS) at different temperatures.**

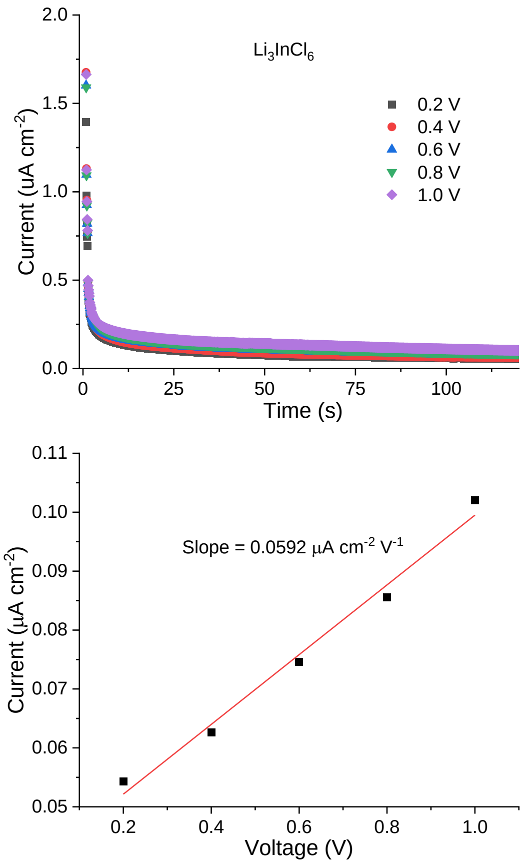


**Supplementary Fig. 23. DC polarization measurements for the $Li_3InCl_6$.** They are tested with the applied voltages of 0.2, 0.4, 0.6, 0.8, and 1.0 V for 2 min and the corresponding current response of the cells at different voltages is calculated.

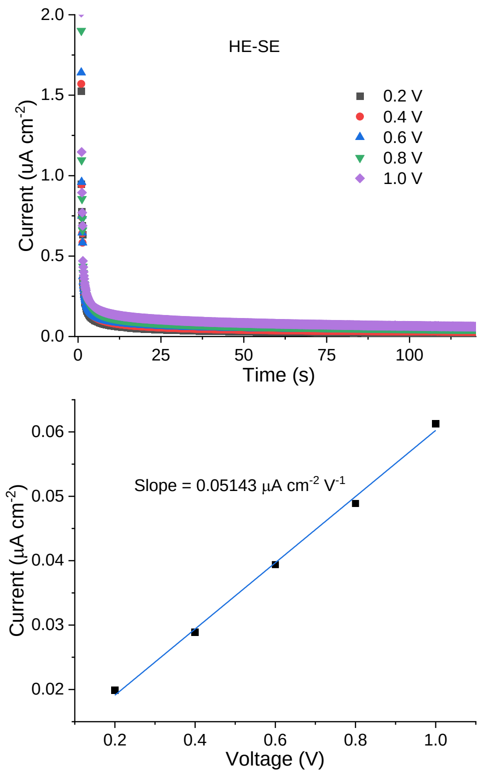


**Supplementary Fig. 24. DC polarization measurements for the HE-SE.** They are tested with the applied voltages of 0.2, 0.4, 0.6, 0.8, and 1.0 V for 2 min and the corresponding current response of the cells at different voltages is calculated.

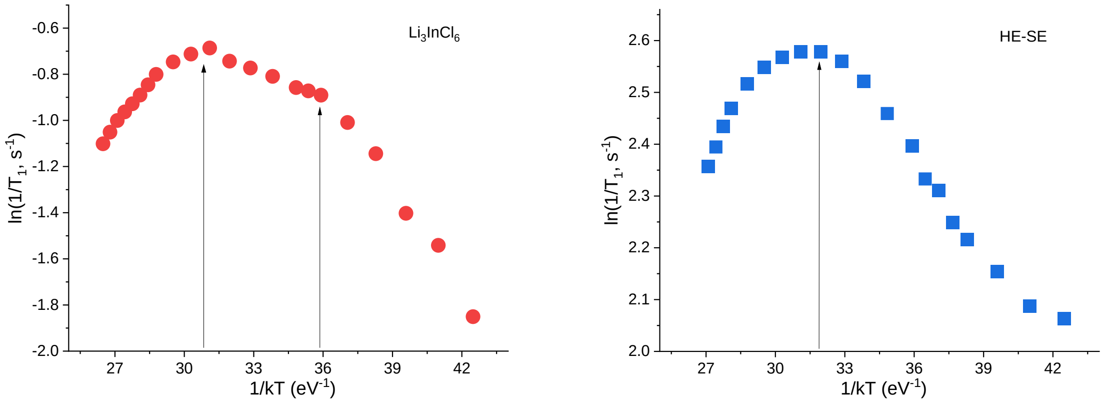


**Supplementary Fig. 25. Temperature-dependent solid-state $^7$Li NMR.** $^7$Li spin−lattice relaxation (SLR) NMR rate measured as a function of temperature for the $Li_3InCl_6$ and the HE-SE. The activation energies of the two SEs are calculated as 0.075±0.021 eV and 0.122±0.011 eV for the HE-SE and $Li_3InCl_6$ at the low-temperature regions, respectively. The results indicate the HE-SE shows an improved Li-ion diffusion and lower Li-ion diffusion energy barrier, which are in good agreement with impedance tests. But it is important to note that the smaller values of the activation energies could be attributed to the exclusion of contributions from grain boundaries, representing a combination of the short-range, local Li-ion motional processes[1,3,6].

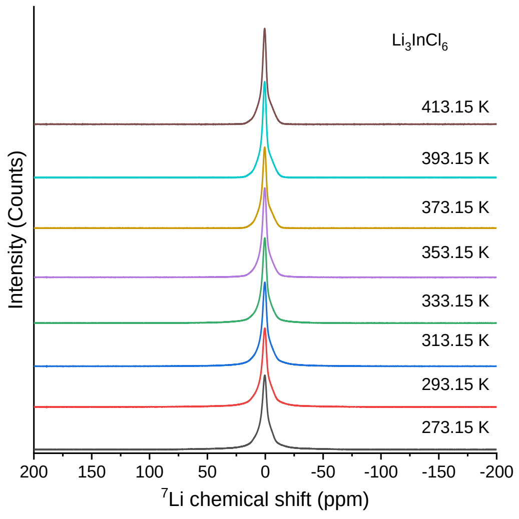


**Supplementary Fig. 26. Curves of the static $^{7}$Li NMR of the general $Li_3InCl_6$.** The temperature range is from 273.15 to 413.15 K.

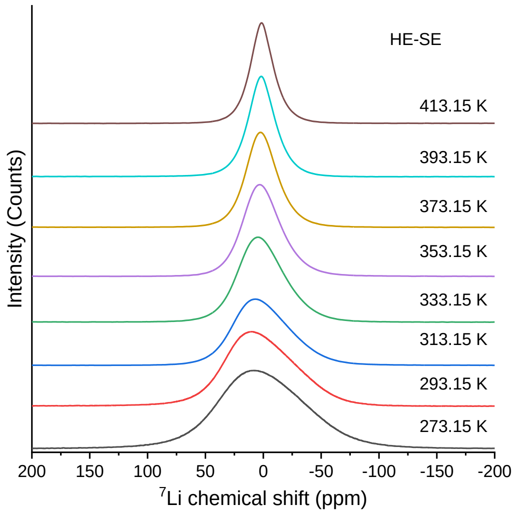


**Supplementary Fig. 27. Curves of the static $^7$Li NMR of the HE-SE.** The temperature range is from 273.15 to 413.15 K. At a temperature of 273.15 K, the asymmetric peak of the static $^7$Li NMR pattern of the HE-SE could suggest that the motion of the $Li^+$ across the different sites does not average out completely. As the temperature increases, the spectra become narrower and more symmetric, reflecting that mobile Li-ions increasingly average out the dipolar interactions[1,2].

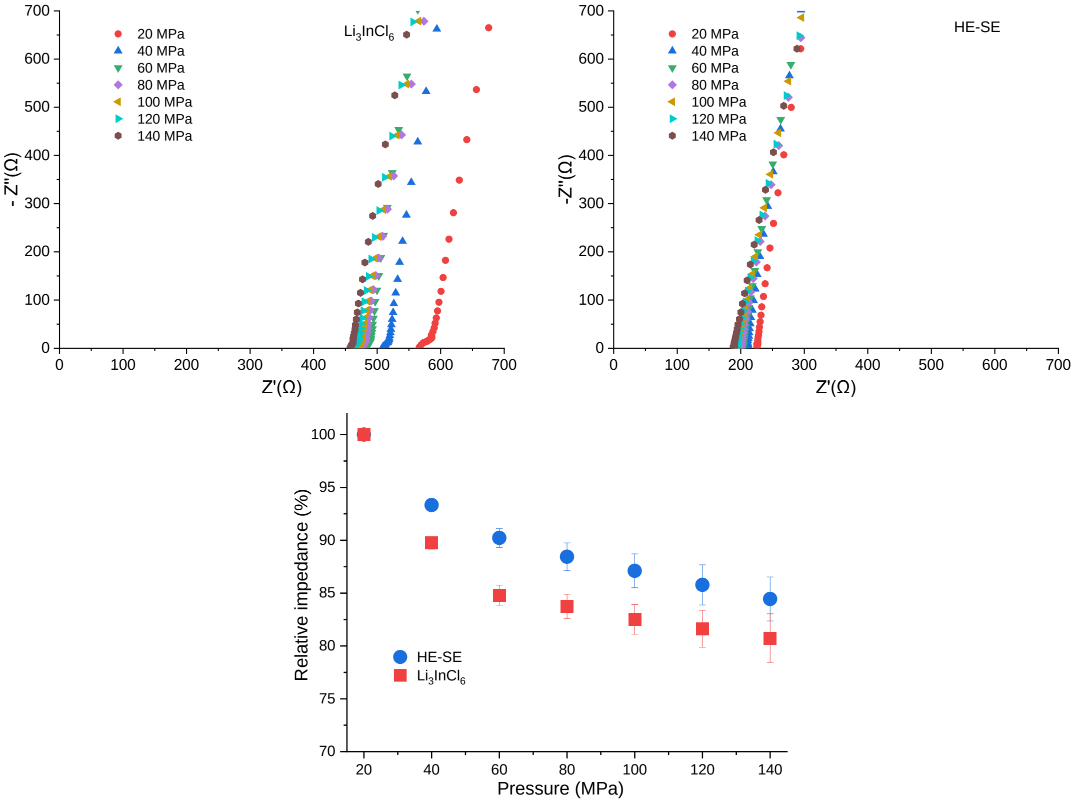


**Supplementary Fig. 28. EIS and relative impedance for $Li_3InCl_6$ and the HE-SE collected at various pressures at room temperature.** The pressure range is from 20 to 140 MPa. A larger amount of electrolyte powder of about 350 mg is used to study the influence of the material's morphology and size. Error bars are obtained by measure three cells.

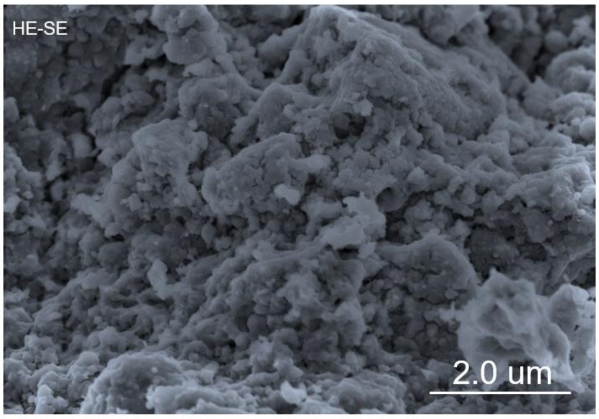


**Supplementary Fig. 29. Morphology characterization the HE-SE electrolyte pellet at the pressure of 80 MPa.** It is from the cross-section view.

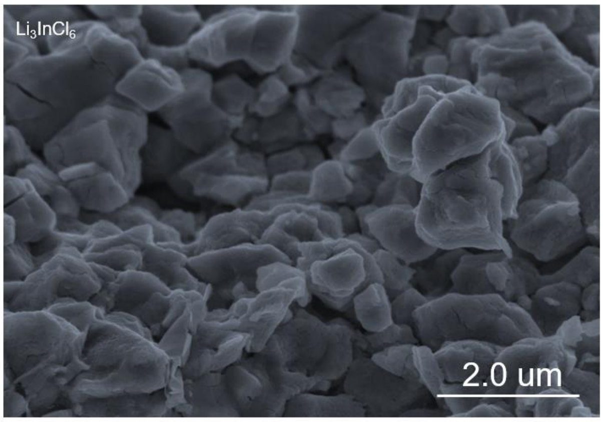


**Supplementary Fig. 30. Morphology characterization the $Li_3InCl_6$ electrolyte pellet at the pressure of 80 MPa.** It is from the cross-section view.

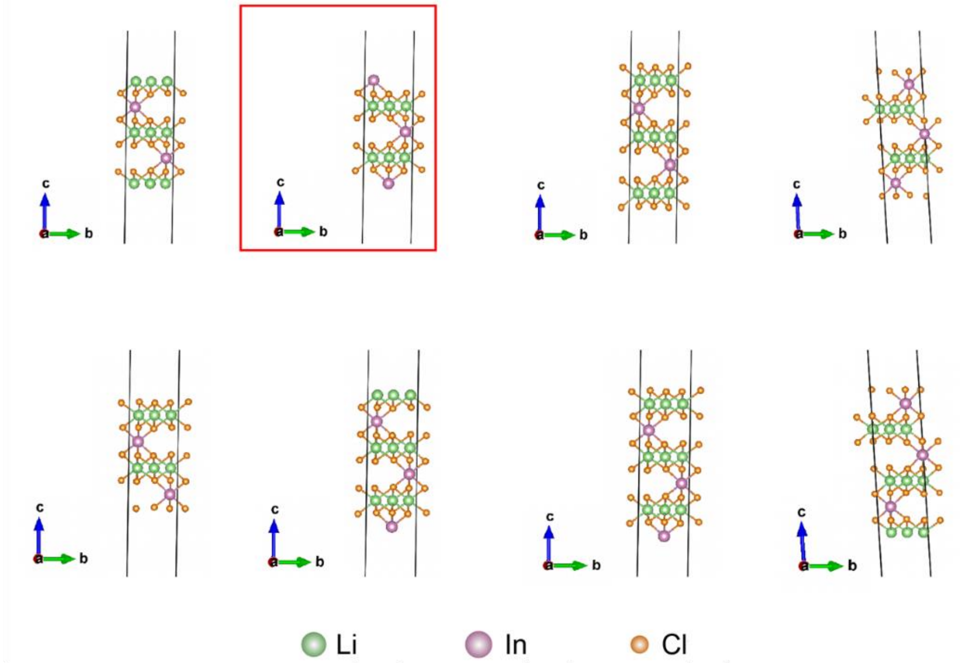


**Supplementary Fig. 31. Surface models of eight (001) orientations of $Li_3InCl_6$ structure.** The model with two $In^{3+}$ terminals in the red rectangle shows the lowest surface energy among models.

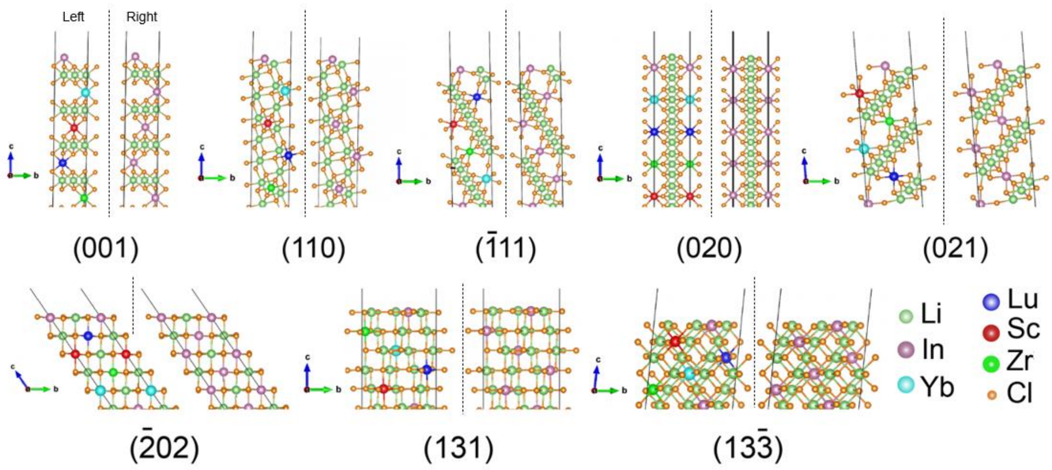


**Supplementary Fig. 32. Surface models of eight orientations of HE-SE (left) and $Li_3InCl_6$ (right) structures.**

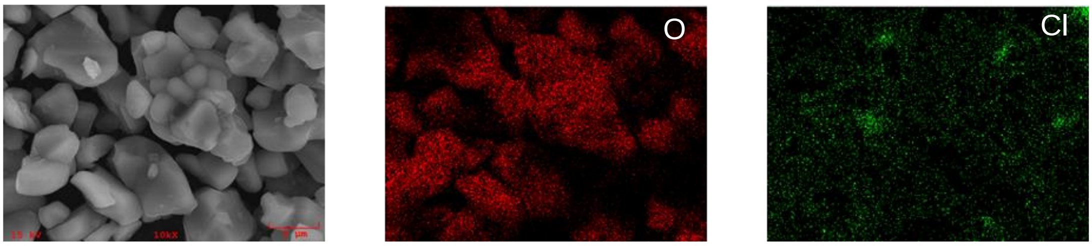


**Supplementary Fig. 33. Electrolyte distribution on NCM cathode.** EDS mappings of O and Cl are used to reflect the distribution of the oxide cathode and HE-SE.

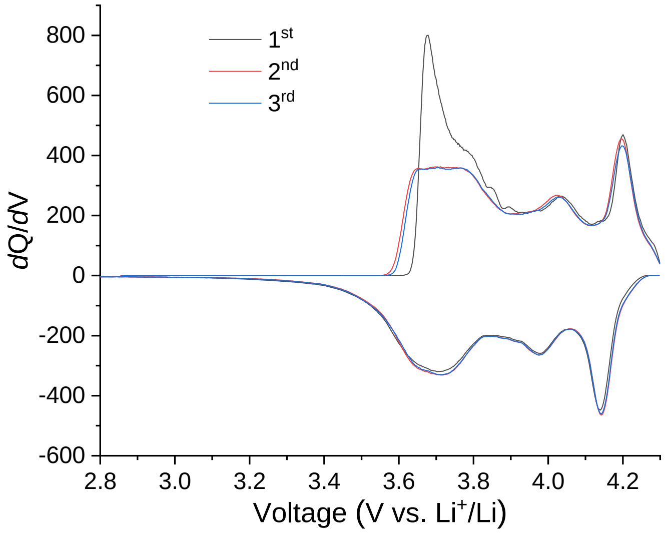


**Supplementary Fig. 34. The corresponding *d*Q/*d*V curves of ASSBs with the HE-SE.** The voltage window is 2.8-4.3 V vs. $Li^+$/Li (2.18-3.68 V vs. $Li^+$/In-Li).

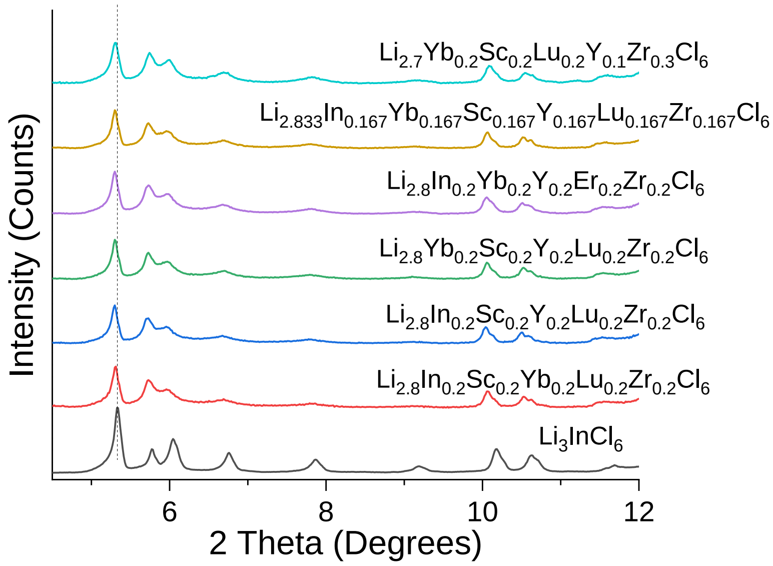


**Supplementary Fig. 35. XRD patterns of HE-SEs.** These representative compositions are obtained based on the analysis of ionic potential, considering the different Me ions and Li content.

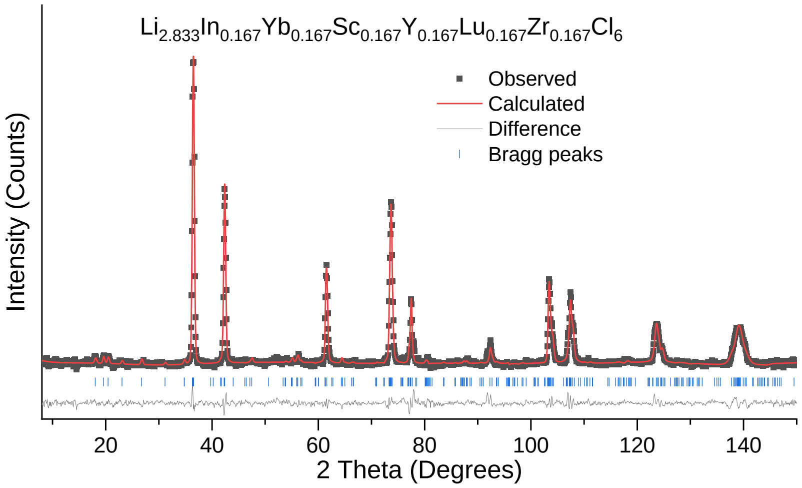


**Supplementary Fig. 36. NPD pattern of $Li_{2.833}In_{0.167}Yb_{0.167}Sc_{0.167}Y_{0.167}Lu_{0.167}Zr_{0.167}Cl_6$.**

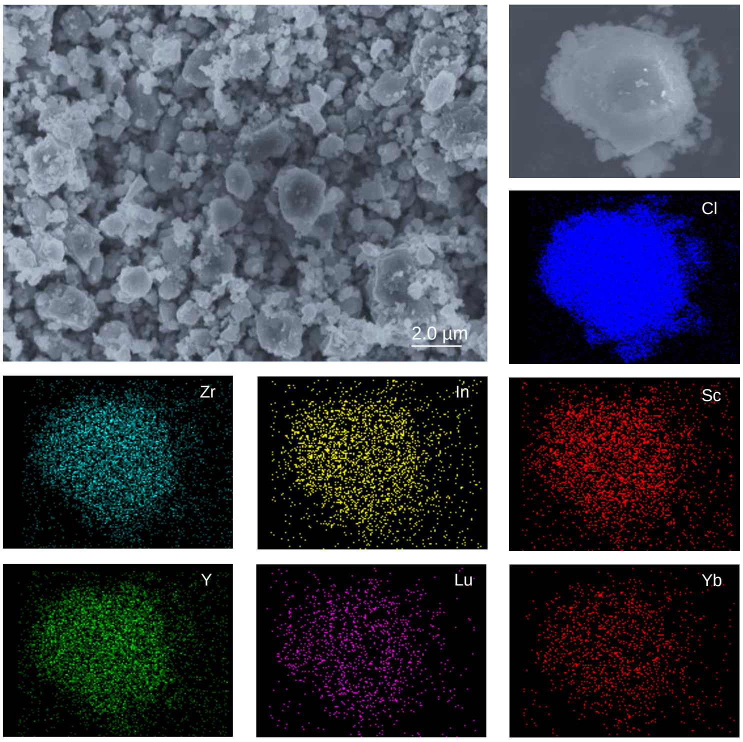


**Supplementary Fig. 37. Morphology and elemental distribution of HE-SE for $Li_{2.833}In_{0.167}Yb_{0.167}Sc_{0.167}Y_{0.167}Lu_{0.167}Zr_{0.167}Cl_6$.**

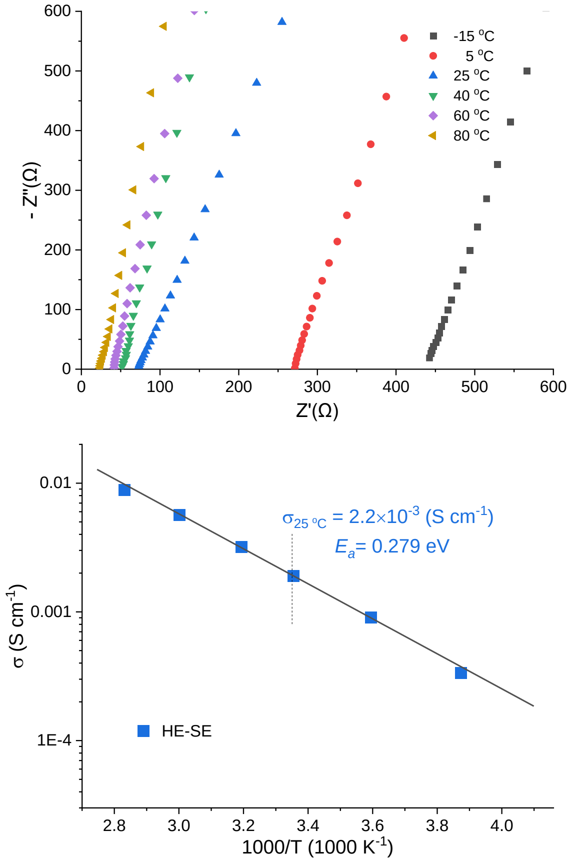


**Supplementary Fig. 38. EIS and corresponding activation energy of six-component HE-SE.**

The composition is $Li_{2.833}In_{0.167}Yb_{0.167}Sc_{0.167}Y_{0.167}Lu_{0.167}Zr_{0.167}Cl_{6}$.

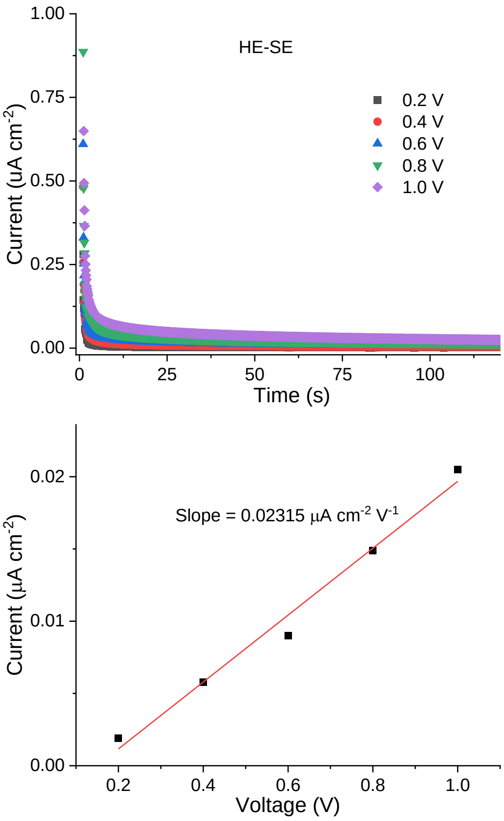


**Supplementary Fig. 39. DC polarization measurements for the as-prepared $Li_{2.833}In_{0.167}Yb_{0.167}Sc_{0.167}Y_{0.167}Lu_{0.167}Zr_{0.167}Cl_6$ HE-SE.** It is tested with the applied voltages of 0.2, 0.4, 0.6, 0.8, and 1.0 V for 2 min and the corresponding current response of the cells at different voltages is calculated.

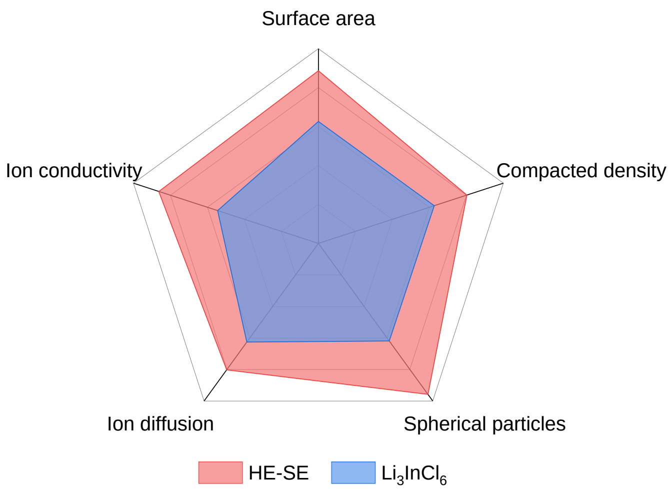


**Supplementary Fig. 40. Comparisons on the $Li_3InCl_6$ and the HE-SE.** They include the materials' morphology/size spherical, compaction density and surface area) and crystal structure (ion diffusion and ion conductivity).

## Supplementary Tables

**Supplementary Table 1. Structural symmetry of Li-Me-X (X = F, Cl, Br and I) compositions.** Me is the metal ions with charge number of 2, 3, 4 and 5, which corresponds to the formal of $Li_2MeX_4$, $Li_3MeX_6$, $Li_2MeX_6$, and $LiMeX_6$, respectively.

| | | *Fd*-3*m* (227) | *P*-3*c*1 (165) | *P*-3*m*1 (164) | *P*-3$_1$*m* (162) | *R*-3 (148) | *Cmmm* (65) | *Pnma* (62) | *Pna*2$_1$ (33) | *C*2/*c* (15) | *P*2$_1$/*c* (14) | *C*2/*m* (12) |
|---|---|---|---|---|---|---|---|---|---|---|---|---|
| | Mg | Cl/Br | | | | | Br | | | | | |
| | Ca | | | | | | | | | | | |
| 2+ | Zn | Cl | | | | | | Cl/Br/I | | | | |
| | Sr | | | | | | | | | | | |
| | Ba | | | | | | | | | | | |
| | Al | | | | | | | | F | F | | |
| | Sc | | F | | | | | | | F | | Cl |
| | Y | | | Cl | | | | Cl | | | | Br |
| | In | | | | | | | | F | | | Cl/Br |
| | Sb | | | | | | | | | | | |
| | La | | | | | | | | | | | |
| | Ce | | | | | | | | | | | |
| | Pr | | | | | | | | | | | |
| | Nd | | | | | | | | | | | |
| | Pm | | | | | | | | | | | |
| 3+ | Sm | | | | | | | | | | | Br |
| | Eu | | | | | | | | | | | Br |
| | Gd | | | | | | | | | | | Br |
| | Tb | | | Cl | | | | | | | | Br |
| | Dy | | | Cl | | | | | | | | Br |
| | Ho | | | Cl | | | | | | | | Br |
| | Er | | | Cl | | | | | | | | Br |
| | Tm | | | Cl | | | | | | | | Br |
| | Yb | | | Cl | | | | Cl | | | | Br |
| | Lu | | | | | | | Cl | | | | Br |
| | Bi | | | | | | | | | | | |
| | Zr | | | Cl | F | | | | | F | F | Cl |
| 4+ | Nb | | | | F | | | | | | | |
| | Hf | | | | F | | | | | | | |
| | Nb | | | | | F | | | | | | |
| 5+ | Sb | | | | | F | | | | | | |
| | Ta | | | | | F | | | | | | |
| | Bi | | | | | F | | | | | | |

**Supplementary Table 2. Calculated weighted average radii of Li and Me ions in monoclinic Li-Me-X phases.**

| | | $\overline{R_{Me}}/\overline{R_X}$ (nm) | $\overline{R_{Me}}$ (nm) | $\overline{R_X}$ (nm) | Ref. |
|---|---|---|---|---|---|
| 1 | $Li_3ScCl_6$ | 0.41160 | 0.0745 | 0.181 | [9] |
| 2 | $Li_3InCl_6$ | 0.44199 | 0.08 | 0.181 | [8] |
| 3 | $Li_2ZrCl_6$ | 0.39779 | 0.072 | 0.181 | [10] |
| 4 | $Li_3In_{0.3}Y_{0.7}Cl_6$ | 0.48066 | 0.086999 | 0.181 | [11] |
| 5 | $Li_3In_{0.9}Y_{0.1}Cl_6$ | 0.44751 | 0.080999 | 0.181 | [11] |
| 6 | $Li_{2.95}In_{0.95}Zr_{0.05}Cl_6$ | 0.43978 | 0.0796 | 0.181 | [12] |
| 7 | $Li_{2.9}In_{0.9}Zr_{0.1}Cl_6$ | 0.43756 | 0.079199 | 0.181 | [12] |
| 8 | $Li_{2.75}In_{0.75}Zr_{0.25}Cl_6$ | 0.43093 | 0.077999 | 0.181 | [12] |
| 9 | $Li_{2.5}In_{0.5}Zr_{0.5}Cl_6$ | 0.41989 | 0.076 | 0.181 | [12] |
| 10 | $Li_3ScBr_6$ | 0.37626 | 0.0745 | 0.198 | [13] |
| 11 | $Li_3YBr_6$ | 0.45455 | 0.09 | 0.198 | [14] |
| 12 | $Li_3InBr_6$ | 0.40404 | 0.08 | 0.198 | [15] |
| 13 | $Li_3SmBr_6$ | 0.48384 | 0.0958 | 0.198 | [14] |
| 14 | $Li_3EuBr_6$ | 0.47828 | 0.0947 | 0.198 | [14] |
| 15 | $Li_3GdBr_6$ | 0.47374 | 0.0938 | 0.198 | [14] |
| 16 | $Li_3TbBr_6$ | 0.46616 | 0.0923 | 0.198 | [14] |
| 17 | $Li_3DyBr_6$ | 0.45960 | 0.091 | 0.198 | [14] |
| 18 | $Li_3HoBr_6$ | 0.45505 | 0.0901 | 0.198 | [14] |
| 19 | $Li_3ErBr_6$ | 0.44949 | 0.089 | 0.198 | [14] |
| 20 | $Li_3TmBr_6$ | 0.44444 | 0.088 | 0.198 | [14] |
| 21 | $Li_3YbBr_6$ | 0.43838 | 0.0868 | 0.198 | [14] |
| 22 | $Li_3LuBr_6$ | 0.43485 | 0.0861 | 0.198 | [14] |
| 23 | $Li_2Mg_{0.5}InBr_6$ | 0.40404 | 0.08 | 0.198 | [16] |
| 24 | $Li_2Ca_{0.5}InBr_6$ | 0.40404 | 0.08 | 0.198 | [16] |
| 25 | $Li_3InCl_{4.8}F_{1.2}$ | 0.47337 | 0.08 | 0.169 | [17] |
| 26 | $Li_3InBr_3Cl_3$ | 0.42216 | 0.08 | 0.1895 | [18] |
| 27 | $Li_3YBr_{4.5}Cl_{1.5}$ | 0.46392 | 0.09 | 0.194 | [19] |
| 28 | $Li_3YBr_3Cl_3$ | 0.47493 | 0.09 | 0.1895 | [19,20] |

**Supplementary Table 3. Calculated weighted average radii of Li and Me ions in hexagonal Li-Me-X compositions.**

| | | $\overline{R_{Me}}/\overline{R_X}$ (nm) | $\overline{R_{Me}}$ (nm) | $\overline{R_X}$ (nm) | Ref. |
|---|---|---|---|---|---|
| 29 | $Li_3YCl_6$ | 0.49724 | 0.09 | 0.181 | [9] |
| 30 | $Li_3TbCl_6$ | 0.50994 | 0.0923 | 0.181 | [9] |
| 31 | $Li_3DyCl_6$ | 0.50276 | 0.091 | 0.181 | [9] |
| 32 | $Li_3HoCl_6$ | 0.49779 | 0.0901 | 0.181 | [9] |
| 33 | $Li_3ErCl_6$ | 0.49171 | 0.089 | 0.181 | [9] |
| 34 | $Li_3TmCl_6$ | 0.48619 | 0.088 | 0.181 | [9] |
| 35 | $Li_3YbCl_6$ | 0.47956 | 0.0868 | 0.181 | [21,22] |
| 36 | $Li_2ZrCl_6$ | 0.39779 | 0.072 | 0.181 | [10] |
| 37 | $Li_{2.9}Y_{0.9}Zr_{0.1}Cl_6$ | 0.48725 | 0.088193 | 0.181 | [23] |
| 38 | $Li_{2.9}Er_{0.9}Zr_{0.1}Cl_6$ | 0.48228 | 0.087293 | 0.181 | [23] |
| 39 | $Li_3YBr_{1.5}Cl_{4.5}$ | 0.48583 | 0.09 | 0.18525 | [19] |

**Supplementary Table 4. Calculated weighted average radii of Li and Me ions in orthorhombic Li-Me-X compositions.**

| | | $\overline{R_{Me}}/\overline{R_X}$ (nm) | $\overline{R_{Me}}$ (nm) | $\overline{R_X}$ (nm) | Ref. |
|---|---|---|---|---|---|
| 40 | $Li_3YCl_6$ | 0.49724 | 0.09 | 0.181 | 9 |
| 41 | $Li_3YbCl_6$ | 0.47956 | 0.0868 | 0.181 | 9 |
| 42 | $Li_3LuCl_6$ | 0.47569 | 0.0861 | 0.181 | 9 |
| 43 | $Li_{2.73}Dy_{1.09}Cl_6$ | 0.54751 | 0.099099 | 0.181 | 24 |
| 44 | $Li_{2.73}Ho_{1.09}Cl_6$ | 0.54209 | 0.098119 | 0.181 | 24 |
| 45 | $Li_{2.73}Er_{1.09}Cl_6$ | 0.53548 | 0.096921 | 0.181 | 24 |
| 46 | $Li_{2.73}Tm_{1.09}Cl_6$ | 0.52946 | 0.095832 | 0.181 | 24 |
| 47 | $Li_{2.633}Y_{0.633}Zr_{0.367}Cl_6$ | 0.46154 | 0.083538 | 0.181 | 23 |
| 48 | $Li_{2.4}Y_{0.4}Zr_{0.6}Cl_6$ | 0.43707 | 0.07911 | 0.181 | 23 |
| 49 | $Li_{2.633}Er_{0.633}Zr_{0.367}Cl_6$ | 0.45804 | 0.082905 | 0.181 | 23 |
| 50 | $Li_{2.4}Er_{0.4}Zr_{0.6}Cl_6$ | 0.43487 | 0.078711 | 0.181 | 23 |
| 51 | $Li_{2.7}Yb_{0.7}Zr_{0.3}Cl_6$ | 0.46304 | 0.083811 | 0.181 | 22 |

**Supplementary Table 5. Ionic radius[25], charge number and ionic potential of different cations in $Li_{3+n}Me_{1+n}X_6$.**

| | Radii (nm) | Valence | $\Phi_{ion}$(nm$^{-1}$) |
|---|---|---|---|
| Li | 0.076 | 1 | 13.15789 |
| Mg | 0.072 | 2 | 27.77778 |
| Ca | 0.100 | 2 | 20.00000 |
| Zn | 0.074 | 2 | 27.02703 |
| Sr | 0.118 | 2 | 16.94915 |
| Ba | 0.135 | 2 | 14.81481 |
| Al | 0.0535 | 3 | 56.07477 |
| Sc | 0.0745 | 3 | 40.26846 |
| Y | 0.09 | 3 | 33.33333 |
| In | 0.08 | 3 | 37.50000 |
| Sb | 0.076 | 3 | 39.47368 |
| La | 0.1032 | 3 | 29.06977 |
| Ce | 0.101 | 3 | 29.70297 |
| Pr | 0.099 | 3 | 30.30303 |
| Nd | 0.0983 | 3 | 30.51882 |
| Pm | 0.097 | 3 | 30.92784 |
| Sm | 0.0958 | 3 | 31.31524 |
| Eu | 0.0947 | 3 | 31.67899 |
| Gd | 0.0938 | 3 | 31.98294 |
| Tb | 0.0923 | 3 | 32.50271 |
| Dy | 0.091 | 3 | 32.96703 |
| Ho | 0.0901 | 3 | 33.29634 |
| Er | 0.089 | 3 | 33.70787 |
| Tm | 0.088 | 3 | 34.09091 |
| Yb | 0.0868 | 3 | 34.56221 |
| Lu | 0.0861 | 3 | 34.84321 |
| Bi | 0.103 | 3 | 29.12621 |
| Zr | 0.072 | 4 | 55.55556 |
| Nb | 0.068 | 4 | 58.82353 |
| Hf | 0.058 | 4 | 68.96552 |
| Nb | 0.064 | 5 | 78.12500 |
| Sb | 0.06 | 5 | 83.33333 |
| Ta | 0.064 | 5 | 78.12500 |
| Bi | 0.076 | 5 | 65.78947 |

**Supplementary Table 6. Ionic radius[25], charge number and ionic potential of possible anions in $Li_{3+n}Me_{1+n}X_6$.**

| Element | Radii (Å) | Valence | $\Phi_{ion}$(nm$^{-1}$) |
|---|---|---|---|
| F | 0.133 | 1 | 7.5188 |
| Cl | 0.181 | 1 | 5.52486 |
| Br | 0.198 | 1 | 5.05051 |
| I | 0.22 | 1 | 4.54545 |
| O | 0.14 | 2 | 14.2857 |

**Supplementary Table 7. Calculated weighted average ionic potential of Li, Me and X ions, as well as the ionic potential ratio for monoclinic Li-Me-X compositions.**

| | $\frac{\overline{\Phi_{Me}}}{\overline{\Phi_{anion}}}$ | $\overline{\Phi_{Li}}$ | $\overline{\Phi_{Me}}$ | $\overline{\Phi_{anion}}$ | Ref. |
|---|---|---|---|---|---|
| $Li_3ScCl_6$ | 1.21477 | 39.47367 | 40.26846 | 33.14916 | 9 |
| $Li_3InCl_6$ | 1.13125 | 39.47367 | 37.5 | 33.14916 | 8 |
| $Li_2ZrCl_6$ | 1.67593 | 26.31579 | 55.55556 | 33.14916 | 10 |
| $Li_3In_{0.3}Y_{0.7}Cl_6$ | 1.04326 | 39.47367 | 34.58331 | 33.14916 | 11 |
| $Li_3In_{0.9}Y_{0.1}Cl_6$ | 1.11867 | 39.47367 | 37.083 | 33.14916 | 11 |
| $Li_{2.95}In_{0.95}Zr_{0.05}Cl_6$ | 1.10192 | 38.4027 | 36.52776 | 33.14916 | 12 |
| $Li_{2.9}In_{0.9}Zr_{0.1}Cl_6$ | 1.18570 | 38.15787 | 39.30501 | 33.14916 | 12 |
| $Li_{2.75}In_{0.75}Zr_{0.25}Cl_6$ | 1.26742 | 36.1842 | 42.01386 | 33.14916 | 12 |
| $Li_{2.5}In_{0.5}Zr_{0.5}Cl_6$ | 1.40387 | 32.8945 | 46.53722 | 33.14916 | 12 |
| $Li_3ScBr_6$ | 1.32886 | 39.47367 | 40.26846 | 30.30306 | 13 |
| $Li_3YBr_6$ | 1.10000 | 39.47367 | 33.33333 | 30.30306 | 14 |
| $Li_3InBr_6$ | 1.23750 | 39.47367 | 37.5 | 30.30306 | 15 |
| $Li_3SmBr_6$ | 1.03340 | 39.47367 | 31.31523 | 30.30306 | 14 |
| $Li_3EuBr_6$ | 1.04541 | 39.47367 | 31.67898 | 30.30306 | 14 |
| $Li_3GdBr_6$ | 1.05544 | 39.47367 | 31.98294 | 30.30306 | 14 |
| $Li_3TbBr_6$ | 1.07259 | 39.47367 | 32.50272 | 30.30306 | 14 |
| $Li_3DyBr_6$ | 1.08791 | 39.47367 | 32.96703 | 30.30306 | 14 |
| $Li_3HoBr_6$ | 1.09878 | 39.47367 | 33.29634 | 30.30306 | 14 |
| $Li_3ErBr_6$ | 1.11236 | 39.47367 | 33.70788 | 30.30306 | 14 |
| $Li_3TmBr_6$ | 1.12500 | 39.47367 | 34.09092 | 30.30306 | 14 |
| $Li_3YbBr_6$ | 1.14055 | 39.47367 | 34.56222 | 30.30306 | 14 |
| $Li_3LuBr_6$ | 1.14982 | 39.47367 | 34.8432 | 30.30306 | 14 |
| $Li_2Mg_{0.5}InBr_6$ | 1.23750 | 40.20384 | 37.5 | 30.30306 | 16 |
| $Li_2Ca_{0.5}InBr_6$ | 1.23750 | 36.31512 | 37.5 | 30.30306 | 16 |
| $Li_3InCl_{4.8}F_{1.2}$ | 1.05509 | 39.47367 | 37.5 | 35.5419 | 17 |
| $Li_3InBr_3Cl_3$ | 1.18199 | 39.47367 | 37.5 | 31.72611 | 18 |
| $Li_3YBr_{4.5}Cl_{1.5}$ | 1.07476 | 39.47367 | 33.33333 | 31.0146 | 19 |
| $Li_3YBr_3Cl_3$ | 1.05066 | 39.47367 | 33.33333 | 31.72611 | 19,20 |

**Supplementary Table 8. Calculated weighted average ionic potential of Li, Me and X ions, as well as the ionic potential ratio for hexagonal Li-Me-X compositions.**

| | $\frac{\overline{\Phi_{Me}}}{\overline{\Phi_{anion}}}$ | $\overline{\Phi_{Li}}$ | $\overline{\Phi_{Me}}$ | $\overline{\Phi_{anion}}$ | Ref. |
|---|---|---|---|---|---|
| $Li_3YCl_6$ | 1.00556 | 39.47367 | 33.33333 | 33.14916 | [9] |
| $Li_3TbCl_6$ | 0.98050 | 39.47367 | 32.50272 | 33.14916 | [9] |
| $Li_3DyCl_6$ | 0.99451 | 39.47367 | 32.96703 | 33.14916 | [9] |
| $Li_3HoCl_6$ | 1.00444 | 39.47367 | 33.29634 | 33.14916 | [9] |
| $Li_3ErCl_6$ | 1.01685 | 39.47367 | 33.70788 | 33.14916 | [9] |
| $Li_3TmCl_6$ | 1.02841 | 39.47367 | 34.09092 | 33.14916 | [9] |
| $Li_3YbCl_6$ | 1.04263 | 39.47367 | 34.56222 | 33.14916 | [21,22] |
| $Li_2ZrCl_6$ | 1.67593 | 26.31579 | 55.55556 | 33.14916 | [10] |
| $Li_{2.9}Y_{0.9}Zr_{0.1}Cl_6$ | 1.07092 | 38.15784 | 35.5 | 33.14916 | [23] |
| $Li_{2.9}Er_{0.9}Zr_{0.1}Cl_6$ | 1.08109 | 38.15784 | 35.8371 | 33.14916 | [23] |
| $Li_3YBr_{1.5}Cl_{4.5}$ | 1.02761 | 39.47367 | 33.33333 | 32.43765 | [19] |

**Supplementary Table 9. Calculated weighted average ionic potential of Li, Me and X ions, as well as the ionic potential ratio for orthorhombic Li-Me-X compositions.**

| | $\frac{\overline{\Phi_{Me}}}{\overline{\Phi_{anion}}}$ | $\overline{\Phi_{Li}}$ | $\overline{\Phi_{Me}}$ | $\overline{\Phi_{anion}}$ | Ref. |
|---|---|---|---|---|---|
| $Li_3YCl_6$ | 1.00556 | 39.47367 | 33.33333 | 33.14916 | 9 |
| $Li_3YbCl_6$ | 1.04263 | 39.47367 | 34.56222 | 33.14916 | 9 |
| $Li_3LuCl_6$ | 1.05110 | 39.47367 | 34.8432 | 33.14916 | 9 |
| $Li_{2.73}Dy_{1.09}Cl_6$ | 1.08391 | 35.92104 | 35.93076 | 33.14916 | 24 |
| $Li_{2.73}Ho_{1.09}Cl_6$ | 1.09474 | 35.92104 | 36.28968 | 33.14916 | 24 |
| $Li_{2.73}Er_{1.09}Cl_6$ | 1.10827 | 35.92104 | 36.73821 | 33.14916 | 24 |
| $Li_{2.73}Tm_{1.09}Cl_6$ | 1.12086 | 35.92104 | 37.15569 | 33.14916 | 24 |
| $Li_{2.633}Y_{0.633}Zr_{0.367}Cl_6$ | 1.25693 | 34.64472 | 41.66601 | 33.14916 | 23 |
| $Li_{2.4}Y_{0.4}Zr_{0.6}Cl_6$ | 1.40777 | 31.57893 | 46.66632 | 33.14916 | 23 |
| $Li_{2.633}Er_{0.633}Zr_{0.367}Cl_6$ | 1.26408 | 34.64472 | 41.90322 | 33.14916 | 23 |
| $Li_{2.4}Er_{0.4}Zr_{0.6}Cl_6$ | 1.41229 | 31.57893 | 46.81614 | 33.14916 | 23 |
| $Li_{2.7}Yb_{0.7}Zr_{0.3}Cl_6$ | 1.23251 | 35.5263 | 40.85676 | 33.14916 | 22 |

**Supplementary Table 10. Calculated weighted average ionic potential of Li, Me and X ions, as well as the ionic potential ratio for $Li_{2.8}In_{0.2}Sc_{0.2}Yb_{0.2}Lu_{0.2}Zr_{0.2}Cl_6$ composition.**

| | $\frac{\overline{\Phi_{Me}}}{\overline{\Phi_{anion}}}$ | $\overline{\Phi_{Li}}$ | $\overline{\Phi_{Me}}$ | $\overline{\Phi_{anion}}$ |
|---|---|---|---|---|
| $Li_{2.8}In_{0.2}Sc_{0.2}Yb_{0.2}Lu_{0.2}Zr_{0.2}Cl_6$ | 1.22314 | 36.84207 | 40.5459 | 33.14916 |

**Supplementary Table 11. Crystallographic and Rietveld refinement data of the as-prepared $Li_{2.8}In_{0.2}Sc_{0.2}Yb_{0.2}Lu_{0.2}Zr_{0.2}Cl_6$ compound.**

| | |
|---|---|
| Space group | *C*2/*m* - monoclinic |
| Wavelengths | 1.886 Å |
| Temperature | ~300 K |
| Cell parameters | $a$ = 6.44058(35) Å |
| | $b$ = 11.1548(4) Å |
| | $c$ = 6.3818(4) Å |
| | $\alpha = \gamma = 90^{o}$, $\beta = 109.6595(16)^{o}$ |
| | $V$ = 431.76(4) $Å^3$ |
| | $Z$ = 2 |
| Reliability factors | $R_{wp}$ = 9.85% |
| | $R_p$ = 7.29% |
| | *Gof* = 1.34 |

**Supplementary Table 12. Atomic coordinates, occupancies and anisotropic displacement parameters (Å$^2$) of the as-prepared $Li_{2.8}In_{0.2}Sc_{0.2}Yb_{0.2}Lu_{0.2}Zr_{0.2}Cl_6$ compound.**

| | x | y | z | Occupancy | $U_{iso}$ | Wyckoff |
|---|---|---|---|---|---|---|
| Li1 | 1/2 | 0 | 1/2 | 0.6853(3) | 0.02358(1) | 2*d* |
| Li2 | 0 | 0.18652(1) | 1/2 | 0.71331(1) | 0.04676(5) | 4*h* |
| Li3 | 0 | 1/3 | 0 | 0.35097(4) | 0.03737(11) | 4*g* |
| Me4[a] | 0 | 1/3 | 0 | 0.06272(3) | 0.03737(11) | 4*g* |
| Me5[a] | 0 | 0 | 0 | 0.87256(1) | 0.04787(2) | 2*a* |
| Cl6 | 0.24761(2) | 0 | -0.23803(2) | 1.0 | 0.01374(7) | 4i |
| Cl7 | 0.2411(31) | 0.16195(2) | 0.2354(5) | 1.0 | 0.01898(3) | 8*j* |

[a]The ratio of Me4 and Me5 are the mixture of $In_{0.2}Sc_{0.2}Yb_{0.2}Lu_{0.2}Zr_{0.2}$.

**Supplementary Table 13. Calculated weighted average ionic potential of Li, Me and X ions, as well as the ionic potential ratio for HE halides.**

| | $\frac{\overline{\Phi_{Me}}}{\overline{\Phi_{anion}}}$ | $\overline{\Phi_{Li}}$ | $\overline{\Phi_{Me}}$ | $\overline{\Phi_{anion}}$ |
|---|---|---|---|---|
| $Li_{2.8}In_{0.2}Sc_{0.2}Y_{0.2}Lu_{0.2}Zr_{0.2}Cl_6$ | 1.21572 | 36.84207 | 40.30014 | 33.14916 |
| $Li_{2.8}Yb_{0.2}Sc_{0.2}Y_{0.2}Lu_{0.2}Zr_{0.2}Cl_6$ | 1.19800 | 36.84207 | 39.71256 | 33.14916 |
| $Li_{2.8}In_{0.2}Yb_{0.2}Y_{0.2}Er_{0.2}Zr_{0.2}Cl_6$ | 1.19800 | 36.84078 | 39.71256 | 33.14916 |
| $Li_{2.833}In_{0.167}Yb_{0.167}Sc_{0.167}Y_{0.167}Lu_{0.167}Zr_{0.167}Cl_6$ | 1.17209 | 37.28067 | 38.85378 | 33.14916 |
| $Li_{2.7}Yb_{0.2}Sc_{0.2}Lu_{0.2}Y_{0.1}Zr_{0.3}Cl_6$ | 1.26502 | 35.52630 | 41.93451 | 33.14916 |

**Supplementary Table 14. Crystallographic and Rietveld refinement data of the as-prepared $Li_{2.833}In_{0.167}Yb_{0.167}Sc_{0.167}Y_{0.167}Lu_{0.167}Zr_{0.167}Cl_6$ compound.**

| | |
|---|---|
| Space group | *C*2/*m* - monoclinic |
| Wavelengths | 1.886 Å |
| Temperature | ~300 K |
| Cell parameters | $a$ = 6.403(24) Å<br>$b$ = 11.07(4) Å<br>$c$ = 6.360(24) Å<br>$\alpha = \gamma = 90^o$, $\beta = 109.279(22)^o$<br>$V$ = 426.1(5) Å$^3$<br>$Z$ = 2 |
| Reliability factors | $R_{wp}$ = 4.95%<br>$R_p$ = 3.73%<br>*Gof* = 3.47 |

**Supplementary Table 15. Atomic coordinates, occupancies and anisotropic displacement parameters (Å$^2$) of the as-prepared $Li_{2.833}In_{0.167}Yb_{0.167}Sc_{0.167}Y_{0.167}Lu_{0.167}Zr_{0.167}Cl_6$ compound.**

| | x | y | z | Occupancy | $U_{iso}$ | Wyckoff |
|---|---|---|---|---|---|---|
| Li1 | 1/2 | 0 | 1/2 | 0.762(1) | 0.02366(2) | 2*d* |
| Li2 | 0 | 0.18567(3) | 1/2 | 0.7961(2) | 0.01263(21) | 4*h* |
| Li3 | 0 | 1/3 | 0 | 0.24232(13) | 0.07767(5) | 4*g* |
| Me4[c] | 0 | 1/3 | 0 | 0.07813(3) | 0.07767(5) | 4*g* |
| Me5[c] | 0 | 0 | 0 | 0.84375(2) | 0.06835(16) | 2*a* |
| Cl6 | 0.25353(1) | 0 | -0.23739(5) | 1.0 | 0.03567(3) | 4i |
| Cl7 | 0.24947(7) | 0.16369(2) | 0.24391(1) | 1.0 | 0.01959(1) | 8*j* |

[c]The ratio of Me4 and Me5 are the mixture of $In_{0.167}Yb_{0.167}Sc_{0.167}Y_{0.167}Lu_{0.167}Zr_{0.167}$.

## Supplementary References


1 Yu, C. *et al.* Unravelling Li-Ion Transport from Picoseconds to Seconds: Bulk versus Interfaces in an Argyrodite $Li_6PS_5Cl–Li_2S$ All-Solid-State Li-Ion Battery. *Journal of the American Chemical Society* **138**, 11192-11201 (2016).

2 Deiseroth, H.-J. *et al.* $Li_6PS_5X$: A Class of Crystalline Li-Rich Solids With an Unusually High $Li^+$ Mobility. *Angewandte Chemie International Edition* **47**, 755-758 (2008).

3 Ganapathy, S., Yu, C., van Eck, E. R. H. & Wagemaker, M. Peeking across Grain Boundaries in a Solid-State Ionic Conductor. *ACS Energy Letters* **4**, 1092-1097 (2019).

4 Wilkening, M. & Heitjans, P. From Micro to Macro: Access to Long-Range $Li^+$ Diffusion Parameters in Solids via Microscopic $^{6,7}Li$ Spin-Alignment Echo NMR Spectroscopy. *ChemPhysChem* **13**, 53-65 (2012).

5 Kuhn, A. *et al.* Li self-diffusion in garnet-type $Li_7La_3Zr_2O_{12}$ as probed directly by diffusion-induced $^7Li$ spin-lattice relaxation NMR spectroscopy. *Physical Review B* **83**, 094302 (2011).

6 Epp, V., Gün, Ö., Deiseroth, H.-J. & Wilkening, M. Highly Mobile Ions: Low-Temperature NMR Directly Probes Extremely Fast $Li^+$ Hopping in Argyrodite-Type Li6PS5Br. *The Journal of Physical Chemistry Letters* **4**, 2118-2123 (2013).

7 Kuhn, A. *et al.* NMR relaxometry as a versatile tool to study Li ion dynamics in potential battery materials. *Solid State Nuclear Magnetic Resonance* **42**, 2-8 (2012).

8 Schmidt, M. O., Wickleder, M. S. & Meyer, G. Zur Kristallstruktur von $Li_3InCl_6$. *Zeitschrift für anorganische und allgemeine Chemie* **625**, 539-540 (1999).

9 Bohnsack, A. *et al.* Ternäre Halogenide vom Typ $A_3MX_6$. VI [1]. Ternäre Chloride der Selten-Erd-Elemente mit Lithium, $Li_3MCl_6$ (M = Tb-Lu, Y, Sc): Synthese, Kristallstrukturen und Ionenbewegung. *Zeitschrift für anorganische und allgemeine Chemie* **623**, 1067-1073 (1997).

10 Wang, K. *et al.* A cost-effective and humidity-tolerant chloride solid electrolyte for lithium batteries. *Nature Communications* **12**, 4410 (2021).

11 Li, X. *et al.* Origin of Superionic $Li_3Y_{1-x}In_xCl_6$ Halide Solid Electrolytes with High Humidity Tolerance. *Nano Letters* **20**, 4384-4392 (2020).

12 Luo, X. *et al.* Heterovalent Cation Substitution to Enhance the Ionic Conductivity of Halide Electrolytes. *ACS Applied Materials & Interfaces* **13**, 47610-47618 (2021).

13 Wang, S. *et al.* Lithium Chlorides and Bromides as Promising Solid-State Chemistries for Fast Ion Conductors with Good Electrochemical Stability. *Angewandte Chemie International Edition* **58**, 8039-8043 (2019).

14 Bohnsack, A., Balzer, G., Güdel, H.-U., Wickleder, M. S. & Meyer, G. Ternäre Halogenide vom Typ $A_3MX_6$. VII [1]. Die Bromide $Li_3MBr_6$ (M=Sm-Lu, Y): Synthese, Kristallstruktur, Ionenbeweglichkeit. *Zeitschrift für anorganische und allgemeine Chemie* **623**, 1352-1356 (1997).

15 Okuda, T. & Yamada, K. Structure and Ionic Conductivity of Halocomplexes of Main Group Metallic Elements Studied by NMR and NQR. *Hyperfine Interactions* **159**, 95-102 (2004).

16 Tomita, Y. *et al.* Li ion conductivity of solid electrolyte, $Li_{3-2x}M_xInBr_6$ (M=Mg, Ca, Sr, Ba). *Solid State Ionics* **174**, 35-39 (2004).

17 Zhang, S. *et al.* Advanced High-Voltage All-Solid-State Li-Ion Batteries Enabled by a Dual-Halogen Solid Electrolyte. *Advanced Energy Materials* **11**, 2100836 (2021).

18 Tomita, Y., Matsushita, H., Kobayashi, K., Maeda, Y. & Yamada, K. Substitution effect of ionic conductivity in lithium ion conductor, $LI_3InBr_{6-x}Cl_x$. *Solid State Ionics* **179**, 867-870 (2008).

19 van der Maas, E. *et al.* Investigation of Structure, Ionic Conductivity, and Electrochemical Stability of Halogen Substitution in Solid-State Ion Conductor $Li_3YBr_xCl_{6-x}$. *The Journal of Physical Chemistry C* **127**, 125-132 (2023).

20 Liu, Z. *et al.* High Ionic Conductivity Achieved in $Li_3Y(Br_3Cl_3)$ Mixed Halide Solid Electrolyte via Promoted Diffusion Pathways and Enhanced Grain Boundary. *ACS Energy Letters* **6**, 298-304 (2021).

21 Park, J. *et al.* Heat treatment protocol for modulating ionic conductivity via structural evolution of $Li_{3-x}Yb_{1-x}M_xCl_6$ (M = $Hf^{4+}$, $Zr^{4+}$) new halide superionic conductors for all-solid-state batteries. *Chemical Engineering Journal* **425**, 130630 (2021).

22 Kim, S. Y. *et al.* Lithium Ytterbium-Based Halide Solid Electrolytes for High Voltage All-Solid-State Batteries. *ACS Materials Letters* **3**, 930-938 (2021).

23 Park, K.-H. *et al.* High-Voltage Superionic Halide Solid Electrolytes for All-Solid-State Li-Ion Batteries. *ACS Energy Letters* **5**, 533-539 (2020).

24 Liang, J. *et al.* A Series of Ternary Metal Chloride Superionic Conductors for High-Performance All-Solid-State Lithium Batteries. *Advanced Energy Materials* **12**, 2103921 (2022).

25 Shannon, R. D. Revised effective ionic radii and systematic studies of interatomic distances in halides and chalcogenides. *Acta Crystallographica Section A* **32**, 751-767 (1976).
